# Quantification of cancer driver mutations in human breast and lung DNA using targeted, error‐corrected CarcSeq

**DOI:** 10.1002/em.22409

**Published:** 2020-09-28

**Authors:** Kelly L. Harris, Vijay Walia, Binsheng Gong, Karen L. McKim, Meagan B. Myers, Joshua Xu, Barbara L. Parsons

**Affiliations:** ^1^ US Food and Drug Administration, National Center for Toxicological Research Division of Genetic and Molecular Toxicology Jefferson Arkansas USA; ^2^ Present address: USA; ^3^ US Food and Drug Administration National Center for Toxicological Research, Division of Bioinformatics and Biostatistics Jefferson Arkansas USA

**Keywords:** ACB‐PCR, cancer risk assessment, carcinogenesis, clonal expansion, next‐generation sequencing

## Abstract

There is a need for scientifically‐sound, practical approaches to improve carcinogenicity testing. Advances in DNA sequencing technology and knowledge of events underlying cancer development have created an opportunity for progress in this area. The long‐term goal of this work is to develop variation in cancer driver mutation (CDM) levels as a metric of clonal expansion of cells carrying CDMs because these important early events could inform carcinogenicity testing. The first step toward this goal was to develop and validate an error‐corrected next‐generation sequencing method to analyze panels of hotspot cancer driver mutations (hCDMs). The “CarcSeq” method that was developed uses unique molecular identifier sequences to construct single‐strand consensus sequences for error correction. CarcSeq was used for mutational analysis of 13 amplicons encompassing >20 hotspot CDMs in normal breast, normal lung, ductal carcinomas, and lung adenocarcinomas. The approach was validated by detecting expected differences related to tissue type (normal vs. tumor and breast vs. lung) and mutation spectra. CarcSeq mutant fractions (MFs) correlated strongly with previously obtained ACB‐PCR mutant fraction (MF) measurements from the same samples. A reconstruction experiment, in conjunction with other analyses, showed CarcSeq accurately quantifies MFs ≥10^−4^. CarcSeq MF measurements were correlated with tissue donor age and breast cancer risk. CarcSeq MF measurements were correlated with deviation from median MFs analyzed to assess clonal expansion. Thus, CarcSeq is a promising approach to advance cancer risk assessment and carcinogenicity testing practices. Paradigms that should be investigated to advance this strategy for carcinogenicity testing are proposed.

## 1 INTRODUCTION

Evaluating the carcinogenic potential of test articles to which humans are exposed is a critical endeavor because cancer is the second leading cause of morbidity and mortality among non‐communicable diseases worldwide (Madia *et al*., 2019). Cancer is driven by both exogenously‐ and endogenously‐induced mutational events (Brown *et al*., 2019). Although the most relevant data for assessing carcinogenicity in humans are studies of human populations, ethical considerations and the long latency period for most human cancers prevents the assessment of the carcinogenic potential of therapeutics in humans as part of drug development (Bourcier *et al*., 2015). Retrospective human studies lack sensitivity due to human genetic variation and the impact of many low‐dose co‐exposures (Bourcier *et al*., 2015).

Obstacles to obtaining human data have resulted in a dependency on the two‐year rodent tumor bioassay (RTB) for assessing the potential carcinogenicity of drugs, chemicals, and physical test articles (Bourcier *et al*., 2015). Even though the RTB is called the gold standard for carcinogenicity testing, it is flawed in several ways (Goodman 2018). RTBs require the use of large numbers of animals. The highest dose tested in a RTB is usually the maximum tolerated dose, which can alter biological processes, generate results that may not be relevant to humans (Cohen 2017), and necessitate low dose extrapolation (Bucher 2000). In some instances, rodents are biologically different from humans in terms of xenobiotic metabolism, tumor cell origin, or pathology of premalignant lesions (Silva Lima and Van der Laan 2000; Thayer and Foster 2007; Oesch and Hengstler 2020). Other obstacles are RTBs cost millions of dollars and may take five or more years to complete (Boorman *et al*., 1994).

**FIGURE 1:**
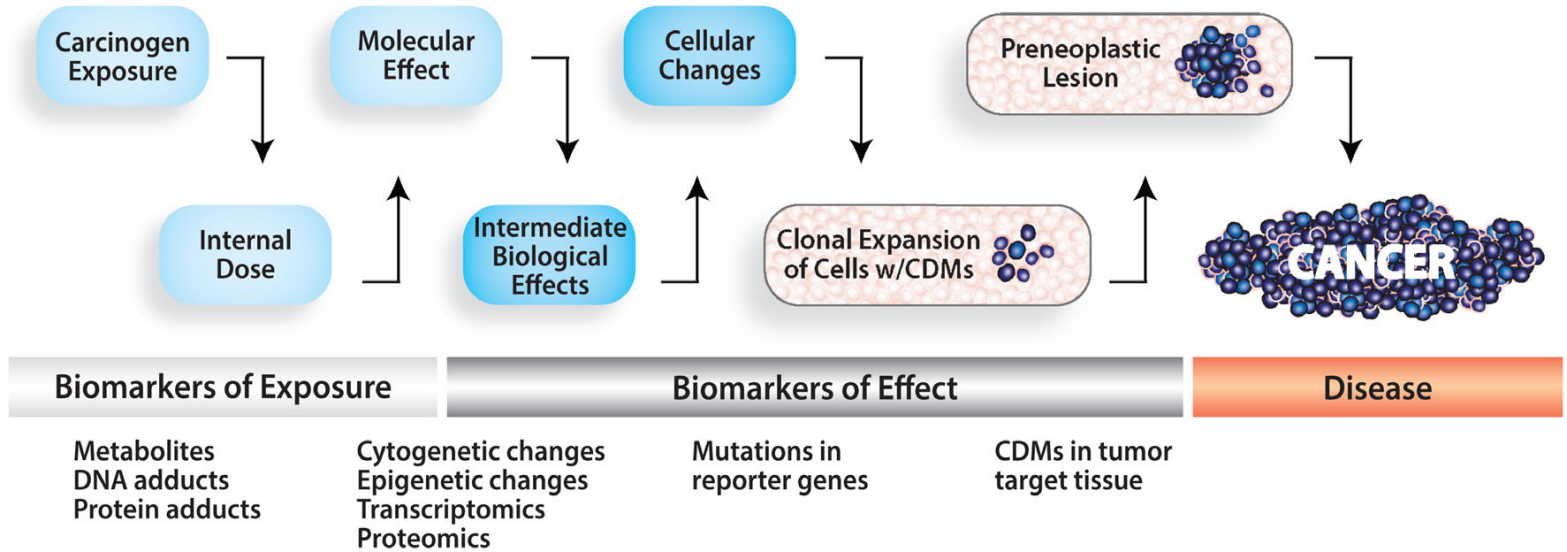
Clonal expansion of cells carrying CDMs is a disease proximate biomarker of effect. A continuum of cancer‐related biomarkers is depicted. Because pathogenic mutations lead to clonal expansion of cells carrying CDMs during carcinogenesis, the variability in CD MF across individuals may be a sensitive metric for assessing cancer risk

Given the drawbacks of the RTB, there is a clear need for in vivo human and rodent biomarkers that can be integrated with other information to predict carcinogenicity (Harris *et al*., 2019). An approach that enables prediction of tumorigenic responses due to lifetime rodent exposures from shorter‐term rodent studies (28 days to 6 months) would be invaluable (Parsons 2018). Hotspot cancer driver mutations (hCDMs) have potential as biomarkers for use in carcinogenicity testing and assessing potential cancer risks associated with exogenous exposures, whether therapeutic, occupational, environmental, genotoxic, or non‐genotoxic (Harris *et al*., 2019). Advantages of hCDMs as biomarkers of cancer risk include their relevance to carcinogenesis in both rodents and humans, their known roles in oncogenesis, and their ability to confer a growth advantage to a neoplastic cell in the microenvironment of the tissue in which the cancer arises, leading to clonal expansion of cells carrying cancer driver mutations (CDMs) (Figure 1) (Stratton *et al*., 2009). hCDMs have been assessed primarily as DNA based measurements, meaning analyses can be performed on any tissue from any species from which DNA can be isolated (Harris *et al*., 2019). When using justifiable assumptions of mutant zygosity, cancer driver (CD) mutant fraction (MF) can be translated into mutant cell numbers or proportions, providing information useful in mathematical modeling of carcinogenesis (Soh *et al*., 2009). Genotoxic carcinogens can induce CDMs, induce other mutations, or epigenetic changes that cooperate with prevalent spontaneous CDMs leading to clonal amplification. Carcinogenesis induced by non‐genotoxic carcinogens is dependent on inducing the clonal expansion of spontaneous CDMs, again detectable through the analysis of prevalent reporter CDMs.

Our lab previously developed an allele‐specific competitive blocker‐polymerase chain reaction (ACB‐PCR, a multi‐step procedure for quantifying levels of specific base pair [bp] substitution mutations in a DNA sample), allowing for quantification of rare mutational events down to a frequency of 10^−5^ (Myers *et al*., 2014b). We used ACB‐PCR to analyze hCDMs across four normal human tissues and established that the degree of interindividual variability in CD MF was positively correlated with the impact of the mutation in terms of organ‐specific carcinogenesis (Parsons *et al*., 2017). This result suggests that hCDMs can serve as substrates for carcinogenesis and reporters of tissue‐specific clonal expansion (Harris *et al*., 2019). ACB‐PCR analyses of carcinogen‐treated rodents demonstrated that variation in CD MF following relatively short‐term exposures (4 weeks to 8 months) correlate with RTB responses (Parsons 2018). While ACB‐PCR was used to generate valuable knowledge regarding the nature of CDMs as biomarkers of cancer risk, it is a low throughput method that assesses one mutation at a time. Given the breadth of possible mutations with importance in tissue‐specific carcinogenesis, a method is needed that can interrogate many hCDMs at once.

Advances in next‐generation sequencing (NGS) have made it possible to analyze many mutations at once, even at low frequency. Indeed, the development of a variety of error‐corrected NGS (EC‐NGS) methods is revolutionizing the field of genetic toxicology (Salk *et al*., 2018), and has created an opportunity to analyze panels of amplicons encompassing many hCDMs. Methods based on the construction of single‐strand consensus sequences (SSCSs) or two‐strand consensus sequences have reported sensitivities between 10^−3^ and 10^−6^ (Kinde *et al*., 2011; Young *et al*., 2015; Gregory *et al*., 2016; McKinzie and Bishop 2020). Duplex sequencing (DS), which is based on constructing double‐strand consensus sequences, is capable of detecting MFs as low as 10^−8^ (Salk *et al*., 2018). Thus, the analysis of panels of hCDMs is now an achievable goal and the development of such panels as biomarkers could be applied to improving carcinogenicity testing.

Although there are advantages to using CDMs as biomarkers for carcinogenicity testing, their development will require progress in multiple areas. First, we must identify which CDMs will be the most useful reporters of carcinogenic effect in different human tissues. Second, we must establish and validate high‐throughput methods for their quantitative analysis. Third, because the planned application is the detection of multiple CDMs in short‐term, repeat‐dose rodent treatment studies, we need to determine which human CDMs are useful reporters of carcinogenic effect in corresponding rodent tissues. This study addressed the first and second areas of necessary research. Specifically, we developed an EC‐NGS method (CarcSeq) for the analysis of a panel of amplicons encompassing human hCDMs in normal and malignant breast and lung samples. We validated the CarcSeq approach in terms of its ability to replicate known tissue specificity, mutation spectra, and concordance with previously obtained ACB‐PCR MF measurements.

## 2 MATERIALS AND METHODS

### 2.1 DNA isolation and multiplex first‐round PCR

DNA was isolated from fresh‐frozen normal breast, normal lung, ductal carcinomas, and lung adenocarcinoma samples, as previously described (Myers *et al*., 2015; Myers *et al*., 2016; Myers *et al*., 2019). Because these samples were purchased from anonymous tissue donors, this work was classified as “not human subjects research,” when evaluated for the purpose of human subject protection. Normal breast and lung were collected as autopsy samples from individuals who died from causes unrelated to sample type. For normal breast, normal lung, ductal carcinomas, and lung adenocarcinomas the mean ± *SD* of tissues processed was 4.12 ± 0.96 g, 2.36 ± 0.64 g, 0.43 ± 0.37 g, and 0.64 ± 0.42 g. Based on the amount of DNA recovered and assuming a diploid genome weight of 6.6 pg (Elli *et al*., 2019), it was calculated that the genomic DNAs analyzed were derived from an average of 2.31 × 10^7^, 6.85 × 10^8^, 1.01 × 10^8^, and 6.56 × 10^7^ diploid cell equivalents for normal breast, normal lung, ductal carcinomas, and lung adenocarcinomas, respectively.

Using 1 μg EcoRI‐digested genomic DNA as template and high‐fidelity *Pfu*Ultra Hotstart DNA Polymerase (Agilent Technologies, Santa Clara, CA), segments of CD genes encompassing hotspot mutations (see Table 1) were amplified from the normal breast (*n* = 9), ductal carcinoma (*n* = 10), normal lung (*n* = 9), and lung adenocarcinoma (*n* = 9) samples. Using the National Center for Biotechnology Information (NCBI) Primer‐BLAST primer selection tool (Ye *et al*., 2012), amplicons 132 bp or less in length encompassing hotspot targets were identified, thereby enabling the entire amplicon to be sequenced using Illumina 150 bp paired‐end sequencing after amplification using primers with 9 bp unique molecular identifier sequences (UMIs) at each end, theoretically generating ~68 billion different 18 bp UMIs. Specifically, four multiplex reactions amplifying two to four gene segments each were performed (Table S1) using primers containing degenerate 5′(N)(N)(N)(N)(N)(N)(N)(N)(N) UMIs. Primers were purchased from Integrated DNA Technologies (Coralville, IA).

**TABLE 1:** hCDMS in amplicons and their COSMIC mutation prevalence in breast ductal carcinomas and lung adenocarcinomas

Multiplex group	Gene	GRCh38 location	Amplicon length/=insert length (bp)	Hotspot codons^a^	Percent total breast ductal carcinomas	Percent total lung adenocarcinomas
	*PIK3CA*‐2	Chr3: 179234268–179234333	126/66	H1047	11.17	0.31
	*KRAS*	Chr12: 25245337–25245377	100/41	G12 G13	0.90 0.19	18.58 1.30
Group 1	*TP53*‐2	Chr17: 7674182–7674266	136/85	G245 R248 R249	0.75 2.87 0.42	1.31 2.20 0.87
	*SETBP1*	Chr18: 44951908–44,951,993	144/86	D868 G870 I871^b^	0.00 0.00 0.00	0.00 0.00 0.00
	*EGFR*‐1	Chr7: 55181323–55181410	145	T790	0.00	1.75
Group 2	*TP53*‐3	Chr17: 7673776–7673848	133/73	R273 P278 R282^c^	3.11 0.38 0.71	2.91 0.47 0.50
	*STK11*	Chr19: 1223086–1223150	122/65	F354^b^	0.00	0.46
	*PIK3CA*‐1	Chr3: 179218270–179218337	131/68	E542 E545	3.01 4.99	0.29 0.69
Group 3	*BRAF*	Chr7: 140753307–140753392	150/86	V600	0.00	1.43
	*TP53*‐1	Chr17: 7675034–7675111	142/78	R175 C176 H179	2.50 0.61 1.08	0.94 0.71 0.55
	*APC*	Chr5: 112839897–112839986	148/90	R1450	0.00	0.00
Group 4	*NFE2L2*	Chr2: 177234022–177234091	131/70	D27^b^	0.00	0.00
	*EGFR‐2*	Chr7: 55191798–55191874	138/77	L858	0.15	3.01

^a^ Hotspot codons identified in Harris *et al*., 2019.

^b^ Hotspot codons identified in COSMIC database.

^c^ Includes the first position of codon R282.

This design produced 13 amplicons covering over 20 hCDMs, flanked by 9 bp UMIs on each end. Primer sequences are provided in Table S1. First‐round PCR reactions were carried out using DNA Engine or DNA Engine Tetrad thermocyclers (Bio‐Rad, Hercules, CA) with the cycling conditions provided in Table S2. All first‐round PCR products were identified by size using gel electrophoresis and purified using the MinElute PCR Purification Kit (Qiagen, Germantown, MD). PCR products were frozen and stored at −80°C, as multiple single‐use aliquots.

### 2.2 Library preparation

**FIGURE 2:**
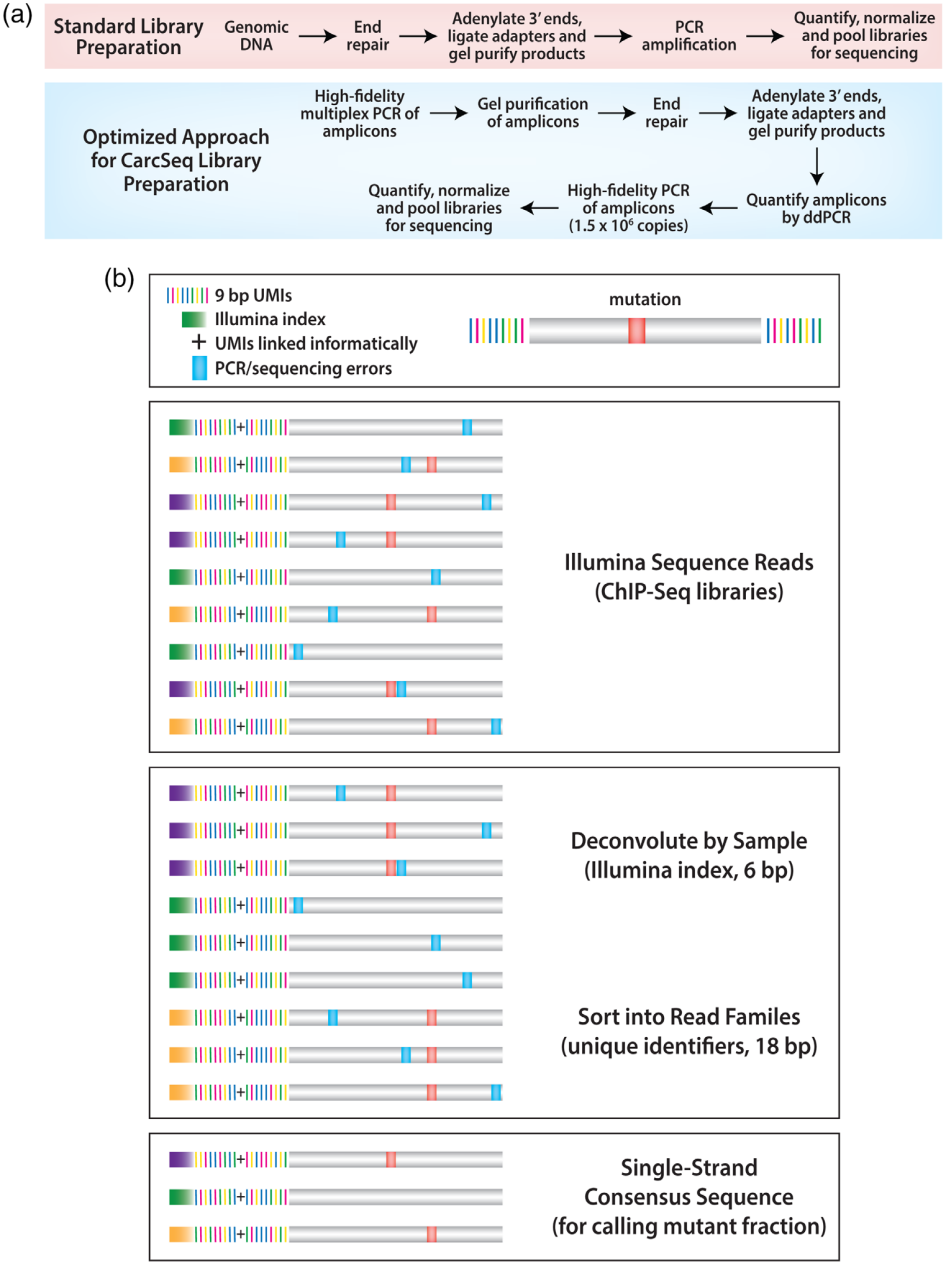
Overview of methods used for EC‐NGS. (a) Standard versus optimized approaches for library preparation are shown. (b) Labeling of amplicons with index and UMIs is used to deconvolute samples and construct SSCSs, respectively, in the CarcSeq method

Libraries composed of combined gel‐purified samples were prepared using the Illumina® TruSeq® ChIP Sample Preparation Kit (Illumina, San Diego, CA), modified for the EC‐NGS application developed in this study. The DNA concentration of each sample was determined by measurements of replicate single‐use aliquots using a dsDNA High Sensitivity Kit (ThermoFisher, Waltham, MA), as previously described (Parsons *et al*., 2010). Multiplex DNA products were combined in equimolar amounts. Following the pooling, 10 ng DNA were subjected to DNA end‐repair, 3′ end adenylation, and ligation of index/adapter sequences, as described for the Illumina® TruSeq® ChIP Sample Preparation Kit (Figure 2a).

Following a second gel purification step to separate target amplicons from unligated adapters, 2 μl of each DNA were serially‐diluted and quantified by digital droplet PCR (ddPCR) using the *PIK3CA* H1047R ddPCR Mutation Detection Assay and the QX200 Droplet Digital PCR System (Bio‐Rad). This information was used to determine the dilution and volume required to incorporate 1 million copies of the *PIK3CA* amplicon for breast or 1.5 million copies of the *PIK3CA* amplicon for lung DNA (and presumably other amplicons) into the final PCR amplification step (18 cycles) of library preparation. This step employed a primer cocktail and PCR master mix to amplify the adapter‐ligated UMI‐labeled amplicons, as per Illumina® TruSeq® PCR kit protocol instructions.

### 2.3 DNA sequencing

Pooled DNA samples were denatured and diluted according to the Illumina® TruSeq® Library Prep Pooling Guide. All DNA samples were diluted to 2 nM and combined with 2 nM PhiX (Illumina). Samples (10 μl) were denatured by the addition of 0.2 N NaOH (10 μl) and diluted with HT1 hybridization buffer to a concentration of 1.8 pM in a total volume of 1.3 ml. Denatured and diluted samples were loaded onto reagent cartridges and cluster generation and sequencing were performed on an Illumina NextSeq 500. In this study, four samples were applied to a mid‐output flow cell and a paired‐end 151‐cycle run was performed on the NextSeq 500, with a 6 bp index read. The NextSeq 500 was controlled using BaseSpace® onsite v2.1 HT. BaseSpace® Real‐Time Analysis (RTA) software extracts intensities from images, performs base calling, and assigns a quality score to each base. Post‐run data processing included sorting reads by sample, based on the 6 bp index sequence incorporated during library preparation (Figure 2b). The post‐sequencing bioinformatics pipeline used is shown in Figure S1 and a detailed description is provided in Supplemental Material, “Error Correction Sequencing Data Processing.” Raw sequencing data have been deposited in the precisionFDA cloud‐based next‐generation DNA sequencing data platform, where access can be provided upon request.

### 2.4 Mutation detection and filtering

Starting with the raw numbers of mutations and the SSCS depths reported for each target base in the mutation position (mutpos) output file, the data were transformed using a three‐step process. First, mutations represented by only one or two mutant SSCSs were removed from the dataset to reduce inaccuracy in MF measurements due to sampling errors (McKinzie *et al*., 2001). Next, for each type of mutation, the MF at each position was calculated (# of mutants [≥3]/# of SSCSs [depth] − # of Ns). The second transformation removed measurements from the data set that were invariant across normal samples because (whether due to real mutational events, uncorrected background errors, artifacts, or SNPs) invariant measurements will not contribute to a biomarker of mutant cell clonal expansion. Specifically, the average MF and MF *SD* were calculated for each type of mutation at each position and a coefficient of variation [COV, (MF *SD*/average MF × 100)] was calculated. Following visualization of known positives and artifacts, invariant MFs were defined as those with a COV <60% and those values were filtered from the normal and tumor data sets. The third data transformation investigated CarcSeq sensitivity by applying cutoffs of either 10^−5^ or 10^−4^, eliminating measurements below the cutoff.

### 2.5 Analysis of CarcSeq sensitivity in a reconstruction experiment

The CarcSeq assay was used alongside ACB‐PCR in a reconstruction experiment analyzing the *PIK3CA* E545K mutation. Mutant and wild type DNAs were prepared from plasmid DNA using a high‐fidelity PCR as previously described (Parsons *et al*., 2017). Wild‐type *PIK3CA* E545 (GAG) and *PIK3CA* E545K mutant (AAG) were mixed to generate samples with MFs of 10^−1^, 10^−2^, 10^−3^, 10^−4^, 10^−5^, and 0 (wild type only). These DNAs were used for library preparation and CarcSeq analysis, as well as ACB‐PCR (Myers *et al*., 2014b). For this analysis, the *PIK3CA* E545K ddPCR Mutant Assay (Bio‐Rad) was used for copy number quantification during library preparation.

### 2.6 Statistical analysis

Comparison of CarcSeq and ACB‐PCR results used log_10_‐transformed MFs. Specifically, Bland–Altman analysis was used to compare MFs quantified by CarcSeq and ACB‐PCR. Correlation analyses were assessed using Pearson's correlation coefficients or Spearman's rank correlation coefficient, for normally distributed and non‐normally distributed data, respectively. Unpaired *t* test with Welch's correction and F tests for equality of variances were used for group comparisons. Statistical analyses were performed using GraphPad Prism 8 Software (GraphPad Software, Inc., La Jolla, CA) with significance defined as *p* ≤ .05 (two‐tailed).

The Adams and Skopek Monte Carlo method was used for comparisons of mutation spectra between sample types (Adams and Skopek 1987).

Variation was analyzed as a metric of clonal expansion using median absolute deviation (MAD) on raw MF measurements as described by Mroz and Rocco (Mroz and Rocco 2013). Specifically, the median MF for MFs ≥10^−4^ in all normal samples (breast or lung, separately) was determined, then the absolute deviation of each measured MF from the overall median was calculated, and the median deviation for each individual normal sample was determined.

A cumulative breast cancer risk was derived from data in the SEER database (https://seer.cancer.gov/data/). Specifically, breast cancer risk at a given age was calculated as the sum of all breast cancer incidence/100,000 for each year of age, up to and including the tissue donor's age.

## 3 RESULTS

### 3.1 CarcSeq cancer panel design and optimization

The overarching goals of this study were to: (a) develop an EC‐NGS method that can quantify many different hCDMs at once, (b) validate measurements of MF based on previous ACB‐PCR measurements obtained from the same samples and expected tissue‐specific mutational profiles and mutation spectra, and (c) garner additional information regarding which mutational targets may serve as tissue‐specific biomarkers of carcinogenic potential.

The Catalogue of Somatic Mutations (COSMIC, a collection of CD gene mutations detected using low‐sensitivity DNA sequencing, https://cancer.sanger.ac.uk/cosmic) was searched to identify the most prevalent mutations in human cancers (Table 1), expecting these to be among the most penetrant mutations and, therefore, appropriate constituents of a multi‐component biomarker. hCDMs that are prevalent in tumors and in normal human tissues may be substrates for chemical carcinogenesis and serve as early reporters of carcinogen‐induced clonal expansion on the path to tumorigenesis (Figure 1) (Brash 2016).

Our search resulted in the identification of 13 gene segments containing hCDMs, which could be subsampled to assess carcinogenicity in different tissues. Target amplification was developed as four multiplex groups, containing two to four amplicons. The multiplex groups, their amplicon targets, GRCh38 locations, lengths, and prevalence in ductal carcinomas and lung adenocarcinomas are provided in Table 1. Table 1 shows that some targets (e.g., *PIK3CA* and *KRAS*) differ in mutation prevalence between tumor types. This study focused on normal and malignant DNA samples from breast and lung because previously obtained ACB‐PCR data (sensitivity 10^−5^) on specific hCDMs within these samples could be used to validate the CarcSeq results. Following amplification of the 13 amplicons, a total of 973 bps of target sequence were generated from each sample, encompassing more than 20 prevalent CDMs (Table 1). This panel was developed to be useful for analysis of multiple tumor types, enabling gene mutations that are not prevalent in breast or lung tumors (Table 1) to serve as negative controls for tissue specificity. When possible, one of the primers was derived from an intronic sequence (sequences shown in blue), to reduce potential confounding due to pseudogene amplification.

First‐round PCR was optimized to ensure the number of PCR duplications was similar to that previously used to generate first‐round PCR products for ACB‐PCR studies that achieved a sensitivity of 10^−5^ (McKinzie and Parsons 2002). The CD gene segments listed in Table 1 were amplified from the normal breast, normal lung, breast ductal carcinoma, and lung adenocarcinoma DNAs. In each case the multiplex products were gel‐purified, quantified, and combined in equimolar amounts.

Libraries were prepared using the Illumina® TruSeq® ChIP Sample Preparation Kit guidance for end‐repair, adenylation of 3′ ends, ligation of indexed paired‐end adapters, and gel‐purification of ligation products (Figure 2a). The next step was to perform a final PCR amplification, converting products with ligated Y‐adapters to double‐strand products containing a sample‐identifying index sequence and the primer‐target bases needed for sequencing. For CarcSeq, this step was modified. Initial experiments used the entire sample obtained after gel‐purification of ligation products to perform final PCR amplification. However, this resulted in populations of sequence reads in which most UMIs were not represented by three reads with the same UMI. This limited the number of SSCSs generated and, therefore, the theoretical sensitivity achievable. We addressed this challenge by reducing input copies before the final PCR step. Specifically, we used ddPCR to quantify the number of *PIK3CA* H1047R molecules in the library and investigated how the use of different numbers of molecules in the final PCR affected recovered SSCS numbers. Limiting the number of molecules in the final PCR to 1–1.5 million maximized the numbers of SSCSs recovered, while generating enough product to complete subsequent sequencing steps.

The number of samples analyzed on an Illumina NSQ 500/550 Mid‐Output flow cell was also optimized. Figure S2 shows that the number of SSCSs obtained per sample decreased as the number of samples applied to the flow cell increased. These values are critical because the total number of SSCSs and the number of molecules in the starting material (3 × 10^5^ copies) defines the theoretical sensitivity of the mutational analysis. Based on this preliminary analysis, four samples (52 amplicons) were analyzed on each mid‐output flow cell. Average and median SSCS numbers achieved for normal, tumor, and all amplicons combined are provided in Table 2, along with the percentages of amplicons analyzed with enough SSCS numbers to achieve theoretical sensitivities of 10^−4^ or 10^−5^ (assuming three molecules are needed to accurately assess MF) and the sensitivity theoretically achievable with 90% power. These calculations are based upon the median SSCS number reported for each amplicon, a number that is often slightly lower at the ends of an amplicon than in the middle. SSCS numbers were larger in the lung data set than in the breast data set. Importantly, the data in Table 2 shows the SSCS numbers obtained robustly support a sensitivity of 10^−4^, with fewer amplicons represented by sufficient SSCS numbers to achieve a sensitivity of 10^−5^. Table 2 shows that sufficient SSCSs were recovered to achieve sensitivities between 2.00 × 10^−5^ and 5.26 × 10^−5^, with 90% power. Bar graphs showing the distribution of SSCS reads for each amplicon are provided in Figure S3. For breast 93% of amplicons had sufficient SSCS representation for a sensitivity of ≥10^−4^. For lung, 96% of amplicons had sufficient SSCS representation for a sensitivity of ≥10^−4^.

**TABLE 2:** Distribution of SSCSs recovered per amplicon and achievable sensitivity

Tissue	Sample type	Average number of SSCSs per amplicon	Median number of SSCSs per amplicon	Percent amplicons with SSCS numbers giving a theoretical sensitivity of 10^−5^	Percent amplicons with SSCS numbers giving a theoretical sensitivity of 10^−4^	Sensitivity with 90% power^a^
Breast	Normal	101,216	87,083	0	89.7	5.26 × 10^−5^
	Ductal carcinoma	130,311	124,753	1.2	96.2	4.09 × 10^−5^
	Combined	116,529	108,855	1.2	93.1	4.57 × 10^−5^
Lung	Normal	267,415	268,424	39.3	98.3	2.00 × 10^−5^
	Adenocarcinoma	170,252	127,959	16.2	94.0	3.13 × 10^−5^
	Combined	218,833	181,279	27.8	96.2	2.44 × 10^−5^

*Note:* This analysis incorporates the requirement to detect three mutant SSCSs to calculate a mutant fraction (i.e., would need ≥300,000 SSCSs to achieve a sensitivity of 10^−5^.

^a^ Calculated using average number of SSCSs per amplicon and StatsToDo: Sample Size for Rare Events Program (http://statstodo.com/SSizRareEvent_Pgm.php).

### 3.2 Analysis and validation of CarcSeq MF measurements

In detection of rare events, sampling error can negatively impact the accuracy of MF measurement (McKinzie *et al*., 2001). Consider the example where 100,000 molecules are assessed, and the true mutation frequency is 1 × 10^−5^. On average, a single mutant molecule should be detected in a sample of 100,000. Due to sampling errors estimated based on a Poisson distribution, the mutation would not be detected in 1/3 of replicate samples (0 mutants sampled) and a MF of 2–5 × 10^−5^ would be detected in 1/3 of replicate samples (a 1–5 fold error in MF) (McKinzie *et al*., 2001). Therefore, we adopted the conservative approach of requiring MFs be based on the detection of at least three mutant DNA molecules (equivalent to three SSCSs).

Our CarcSeq method generated some visually‐identifiable invariant MFs (see Figure S4, panels a–c), one of these being a SNP. Others were the result of reproducible bioinformatic mis‐priming of amplicons with homologous pseudogene sequences (i.e., the MF described the frequency of misalignment, rather than the fraction of mutant molecules in the population, see Figure S4, panel d). Genomic DNA was assessed for some of these putative mutations by ddPCR, which confirmed they were artifacts. Mutations that are invariant across samples are not expected to be useful reporters of carcinogenic effect because carcinogenesis is stochastic in nature (i.e., different sets of events occur in different individuals) and the mutations that induce clonal expansion are expected to vary between samples. To remove invariant artifacts and enrich for mutations associated with clonal expansion, we employed a filter based on COV (MF *SD*/average MF × 100) across normal breast or normal lung samples. The COV for mutations of all types observed across all locations was visualized, along with ACB‐PCR concordant true positives and ddPCR‐confirmed artifacts (see Figure S5 and Table 3). Based on this analysis, only mutations at positions that showed a COV of at least 60% were analyzed further.

**TABLE 3:** Justification for selecting a coefficient of variation cutoff of ≥60% to remove invariant MF measurements and a SNP from the CarcSeq data

Amplicon panel position	GRCh38 location	Gene	Clinvar annotation	Mutation type	Supporting analyses; conclusion	Geometric mean MFs from raw MF data	COV in normal breast (%)	COV in normal lung (%)
105	179218304	*PIK3CA*	Pathogenic E545A‐P	A → C	Reads map to a highly‐homologous sequence outside panel; non‐conserved bases identified in a pseudogene amplified at low frequency	Breast 1.43 × 10^−1^ Lung 1.66 × 10^−1^	7.16	41.45
546	25245337	*KRAS*	Not reported	C → T	Reads map to a highly‐homologous sequence outside panel; non‐conserved bases identified in a pseudogene amplified at low frequency amplified at low frequency	Breast 3.85 × 10^−2^ Lung 9.47 × 10^−2^	21.66	30.17
551	25245342	*KRAS*	Not reported	C → T	Reads map to a highly‐homologous sequence outside panel; non‐conserved bases identified in a pseudogene amplified at low frequency amplified at low frequency	Breast 3.61 × 10^−2^ Lung 9.05 × 10^−2^	23.90	31.38
557	25245348	*KRAS*	Pathogenic G13S‐P	C → T	Shown not to be present in input genomic DNA by ddPCR; a relatively high‐frequency invariant artifact	Breast 1.97 × 10^−2^ Lung 4.60 × 10^−2^	27.42	44.84
707	7674229	*TP53*	Pathogenic G245D‐P	C → T	Shown not to be present in input genomic DNA by ddPCR; a relatively high‐frequency invariant artifact	Breast 6.39 × 10^−3^ Lung 7.04 × 10^−3^	14.14	18.12
342	55181370	*EGFR*	Benign SNP	G → A	16/18 MFs >0.5 at position of a known SNP; reference contains a SNP not in any samples	Breast 6.85 × 10^−1^ Lung 2.23 × 10^−1^	30.57	64.09
104	179218303	*PIK3CA*	Pathogenic E545K	G → A	CarcSeq‐ACB‐PCR concordance at this site 0.92/1.00 for breast/lung; true somatic mutations	Breast 9.08 × 10^−5^ Lung 8.98 × 10^−4^	172.89	109.15
168	179234297	*PIK3CA*	Pathogenic H1047R	A → G	CarcSeq‐ACB‐PCR concordance at this site 0.89/0.85 for breast/lung; true somatic mutations	Breast 5.41 × 10^−3^ Lung 4.18 × 10^−3^	245.29	93.88
559	25245350	*KRAS*	Pathogenic G12D	C → T	CarcSeq‐ACB‐PCR concordance at this site 0.52/0.50 for breast/lung; true somatic mutations	Breast 1.86 × 10^−4^ Lung 3.06 × 10^−4^	81.93	158.32
559	25245350	*KRAS*	Pathogenic G12V‐P	C → A	CarcSeq‐ACB‐PCR concordance at this site 0.51/0.60 for breast/lung; true somatic mutations	Breast 5.70 × 10^−4^ Lung 1.52 × 10^−3^	78.21	69.92

CarcSeq data were analyzed in several ways to ascertain the validity of the measured MFs. CarcSeq validation included testing the expectations that tumors: (a) would have overall higher MFs and greater variability due to more clonal expansion than the corresponding normal samples, (b) would show tissue‐specific differences in types of driver mutations, and (c) would show the reported mutation spectra for breast and lung cancers. Also, CarcSeq measurements should replicate previously obtained ACB‐PCR MF measurements. To investigate the sensitivity of CarcSeq, and the impact of COV filtering, CarcSeq MF measurements were analyzed with and without COV filtering, assuming MF cutoffs of 10^−5^ or 10^−4^.

Table 4 shows that for COV unfiltered MFs two of four comparisons (between normal and tumor for breast and lung using a cutoff of 10^−4^ or 10^−5^) demonstrated a significant increase in tumor geometric mean MF compared with normal geometric mean MF and two of four showed significantly increased variation in tumors as compared with normal. For COV filtered MFs, one of four comparisons showed a significant increase in tumor geometric mean MF compared with normal geometric mean MF and three of four comparisons showed significantly increased variation in tumors as compared with normal. Applying a cutoff of 10^−5^, two of four comparisons (between normal and tumor for breast and lung with and without the COV filter) demonstrated a significant increase in tumor geometric mean MF compared with normal geometric mean MF and one of four showed significantly increased variation in tumors as compared with normal. Applying a cutoff of 10^−4^, one of four comparisons demonstrated a significant increase in tumor geometric mean MF compared with normal geometric mean MF and three of four showed significantly increased variation in tumors as compared with normal.

**TABLE 4:** Summary of mutations detected in normal breast, ductal carcinomas, normal lung, and lung adenocarcinoma samples

	Sample analyzed	Number of mutated positions	Total number of mutations	Geometric mean MF^a^	Unpaired t test with Welch's correction	Variance	F test to compare variances
Breast (>10^−5^)	Normal (all)	886	7,724	7.19 × 10^−5^	***p* < .0001**	0.5024	***p* = .2425**
	Ductal carcinoma (all)	881	9,932	5.77 × 10^−5^		0.5087	
	Normal (COV >0.6)	857	5,500	4.92 × 10^−5^	***p* < .0001**	0.3544	***p* < .0001**
	Ductal carcinoma (COV >0.6)	774	6,353	4.02 × 10^−5^		0.3297	
Lung (>10^−5^)	Normal (all)	937	11,580	4.37 × 10^−5^	***p* < .0001**	0.4822	***p* = .0913**
	Adenocarcinoma (all)	897	10,078	5.34 × 10^−5^		0.4901	
	Normal (COV >0.6)	860	5,850	2.98 × 10^−5^	***p* < .0001**	0.0008	***p* < .0001**
	Adenocarcinoma (COV >0.6)	783	4,655	3.71 × 10^−5^		0.4183	
Breast (>10^−4^)	Normal (all)	490	2,398	2.51 × 10^−4^	***p* < .0001**	0.4957	***p* < .0001**
	Ductal carcinoma (all)	328	2,138	2.90 × 10^−4^		0.5535	
	Normal (COV >0.6)	373	940	1.77 × 10^−4^	***p* = .3520**	0.3192	***p* < .0001**
	Ductal carcinoma (COV >0.6)	177	537	1.85 × 10^−4^		0.5040	
Lung (>10^−4^)	Normal (all)	341	2042	2.64 × 10^−4^	***p* = .6665**	0.4825	***p* < .0001**
	Adenocarcinoma (all)	386	2,141	2.60 × 10^−4^		0.5286	
	Normal (COV >0.6)	164	431	2.23 × 10^−4^	***p* = .1631**	0.4146	***p* < .0001**
	Adenocarcinoma (COV >0.6)	164	470	2.49 × 10^−4^		0.5997	

^a^ MFs = 0 were assigned a MF of 1 × 10^−6^ for geometric mean calculation.

**FIGURE 3:**
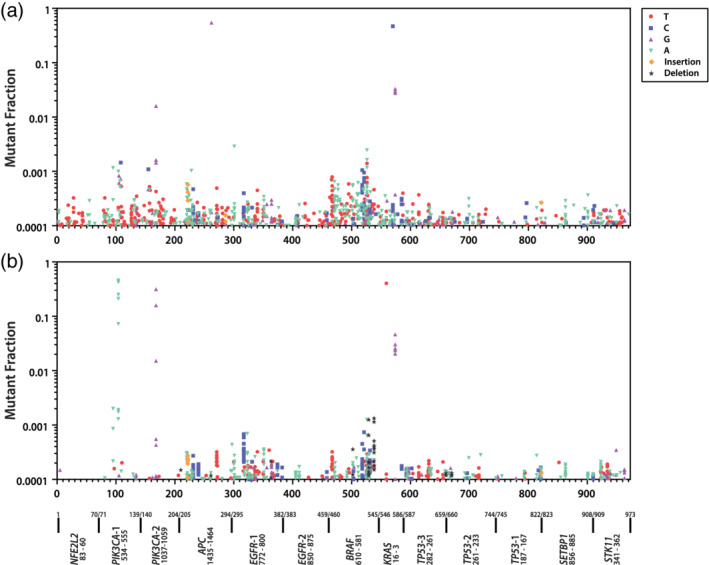
Frequency and distribution of mutations across amplicon targets for normal breast (a) and breast ductal carcinoma (b) samples

**FIGURE 4:**
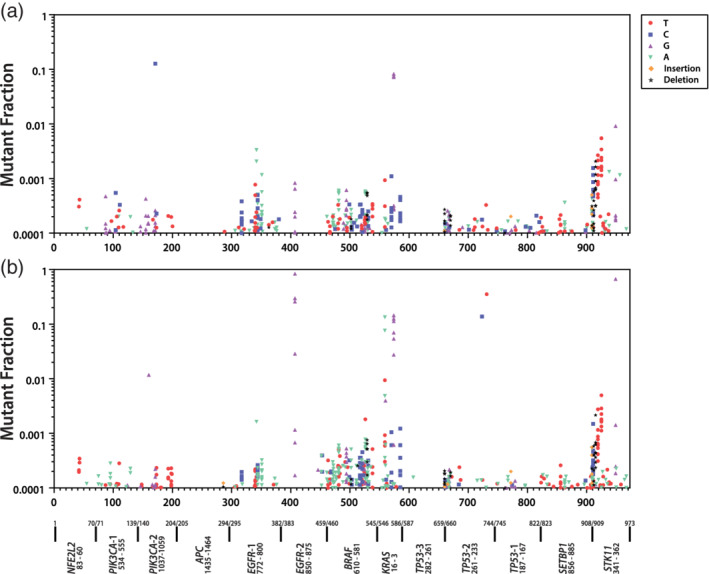
Frequency and distribution of mutations across amplicon targets for normal lung (a) and lung adenocarcinoma (b) samples

In Figures 3 and 4, MF measurements ≥10−4, with COV >60% are plotted above their positions in the 973 bp composite CarcSeq target, arrayed from smallest chromosome number and location to the largest (x‐axis, left to right). Figure 3 shows mutation occurred at a larger number of positions for normal breast (Figure 3a) than for ductal carcinomas (Figure 3b), whereas the ductal carcinomas had more large MF measurements (clonal expansion) at hotspots. The median, mean, and 90th percentile for numbers of mutations at specific target positions were 2, 3.03, and 7 for ductal carcinomas and 2, 2.52, and 5 for normal breast, respectively. And, 2.8% of ductal carcinomas mutations had MFs >3 × 10^−5^ compared with 0.6% of normal breast mutations. Figure 4 shows the composite mutational profiles of the 9 normal lung (Figure 4a) and 10 lung adenocarcinoma samples (Figure 4b) with COV >60% and MFs ≥10^−4^. Normal lung and lung adenocarcinomas had similar numbers of mutated sites. However, a slightly larger number of mutations were found at hotspot positions in lung adenocarcinomas compared with the normal lung; the median, mean, and 90th percentile for numbers of mutations at specific target positions were 2, 2.87, and 7 for lung adenocarcinomas and 2, 2.63, and 7 for normal lung, respectively. A larger percentage of lung adenocarcinomas had MFs >3 × 10^−5^ compared with normal lung (3.6 vs. 1.6%). One unexpected observation was that normal lung and lung adenocarcinomas had similar levels of *STK11* hotspot mutations.

A comparison of Figure 3b and Figure 4b shows that ductal carcinomas contain hotspots and large MF measurements for *PIK3CA* mutation, whereas lung adenocarcinomas show hotspots and large MF measurements in *EGFR*, *KRAS*, *TP53*, and *STK11*. Although *KRAS* mutations were detected in ductal carcinomas, where they accounted for 12/537 (2%) of recovered mutations, *KRAS* mutations were more prevalent in lung adenocarcinomas, where they accounted for 58/470 (12%) of recovered mutations. Therefore, the mutational hotspots match the known impact of the different mutations in the two tumor types (Harris *et al*., 2019).

The mutation spectra observed within the different tissue types were compared, using the COV ≥60% MFs ≥10^−4^ data sets (Table 5). Significant differences in mutation spectra of normal and tumor were observed for both breast and lung. Significant differences in mutation spectra were observed between normal breast and normal lung, as well as between ductal carcinomas and lung adenocarcinomas. Most importantly, the most predominant mutational specificity in each tumor matched that previously reported, G:C → A:T for ductal carcinomas and G:C → T:A for lung adenocarcinomas (Kandoth *et al*., 2013).

**TABLE 5:** Analysis of mutation spectra across sample types

	Breast		Lung	
	Normal	Ductal carcinoma	Normal	Adenocarcinoma
Transitions	Number of mutations (%)		Number of mutations (%)	
G:C → A:T	179 (20)	180 (34)	99 (20)	95 (20)
A:T → G:C	123 (13)	113 (21)	87 (17)	73 (15)
Transversions				
G:C → T:A	468 (51)	135 (25)	98 (20)	175 (37)
G:C → C:G	15 (2)	18 (3)	36 (7)	33 (7)
A:T → C:G	38 (4)	21 (4)	36 (7)	20 (4)
A:T → T:A	80 (9)	34 (6)	32 (6)	38 (8)
Total	910 (100)	537 (100)	503 (100)	478 (100)
Normal vs. tumor	*p* = .0000		*p* = .0000	
Normal vs. Normal	*p* = .0000			
Tumor vs. tumor	*p* = .0000			

### 3.3 Sensitivity

**FIGURE 5:**
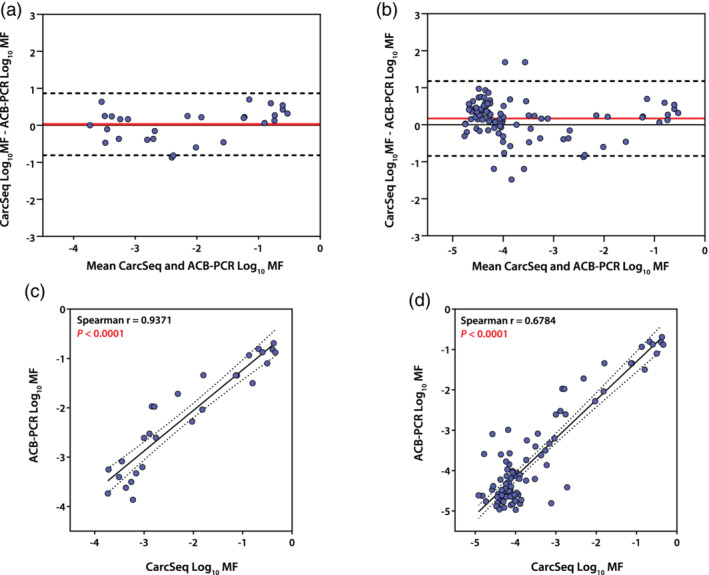
Comparison of CarcSeq and ACB‐PCR MF measurements. Bland–Altman plots of combined breast and lung samples illustrate little bias between CarcSeq and ACB‐PCR MF measurements when both are ≥10^−4^, bias = 0.02883 ± 0.4279, 95% limits of agreement = −0.8098 − 0.8674 (a) and ≥10^−5^ bias = 0.1700 ± 0.5155, 95% limits of agreement = −0.8404 − 1.180 (b). Linear regression analysis of combined breast and lung samples shows high concordance between CarcSeq and ACB‐PCR MF measurements when both are ≥10^−4^ (c) or ≥10^−5^ (d)

To further probe the sensitivity achieved in the CarcSeq analysis of MF and how that relates to the accuracy of MF measurement, a subset of CarcSeq measurements were compared directly with ACB‐PCR measurements performed on the same samples. Specifically, comparisons of all CarcSeq *KRAS* G12D, *KRAS* G12V, *PIK3CA* E545K, and *PIK3CA* H1047 MF measurements ≥10^−4^ or ≥10^−5^ in breast and lung were compared with ACB‐PCR MF measurements (Figure 5c,d, respectively). Bland–Altman showed there was almost no bias between CarcSeq measurements ≥10^−4^ and ACB‐PCR MF measurements (Figure 5a) and only a slight bias in terms of CarcSeq measurements ≥10^−5^ having greater MFs than ACB‐PCR MF measurements (Figure 5b). Spearman correlation analysis determined that, using either cutoff, the CarcSeq and ACB‐PCR MF measurements correlated significantly (*p* < .0001), although the correlation was stronger when MFs ≥10^−4^ were considered (CarcSeq MFs ≥10^−4^, *n* = 28 and Spearman *r* = 0.9371 [linear regression is shown in Figure 5c], whereas for CarcSeq MFs ≥10^−5^, *n* = 95 and Spearman *r* = 0.6784 [linear regression is shown in Figure 5d]). The same type of comparison of CarcSeq and ACB‐PCR MF measurements was performed using the breast or lung MFs separately (Figures S6 and S7, respectively).

**FIGURE 6:**
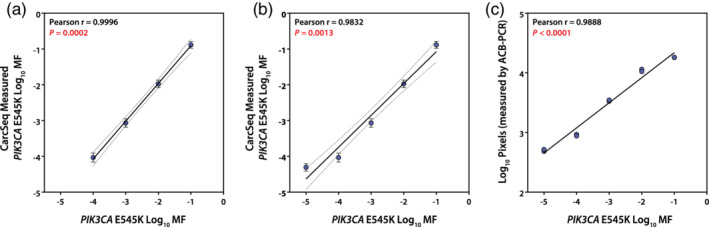
Define mixtures of *PIK3CA* E545K wild type and mutant were analyzed by CarcSeq and ACB‐PCR. The standards analyzed had MFs of 10^−1^, 10^−2^, 10^−3^, 10^−4^, and 10^−5^. The relationship between expected and CarcSeq‐measured MFs ≥10^−4^ is shown in (a). The relationship between expected and CarcSeq‐measured MF ≥10^−5^ is shown in (b). ACB‐PCR analysis of the *PIK3CA* E545K MF standards is shown in (c)

As a direct approach for analyzing CarcSeq sensitivity, a reconstruction experiment was performed. PCR products amplified from plasmid DNAs encompassing the *PIK3CA* E545 wild‐type sequence (GAG) or the E545K mutant (AAG) were mixed in known proportions (10^−1^ to 10^−5^). These DNAs were used for CarcSeq library preparation and standard ACB‐PCR analysis. The reconstruction experiment demonstrated that CarcSeq MF measurements correlated perfectly with the expected MFs in the standards down to a MF of 10^−4^ (Figure 6a), but slightly overestimated the 10^−5^ MF standard (Figure 6b). The ACB‐PCR results analyzing the same set of MF standards is provided for comparison in Figure 6c.

### 3.4 Correlation with age

**FIGURE 7:**
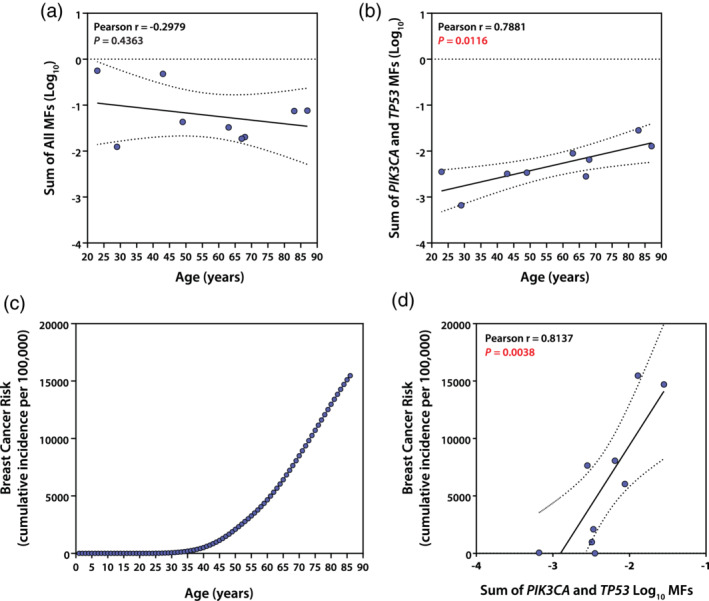
Relationships between the sum of MF measurements in normal breast of different individuals and tissue donor age. The sum of MFs for different individual samples was correlated with age using MFs ≥10^−4^ for all targets (a) or only targets known to be drivers of breast cancer, *PIK3CA* and *TP53* (b). SEER breast cancer incidence data was used to calculate a cumulative risk for each age (c), as the cumulative sum of incidence observed at the current and all previous years. Finally, the sum of *PIK3CA* and *TP53* MFs ≥10^−4^ in normal breast were plotted relative to the cumulative risk expected based on the tissue donor's age (d)

Variation in MF for drivers with tissue‐specific carcinogenic effect is expected to increase with increasing tissue donor age because age is the most important risk factor for cancer. Figure 7 shows that the sum of MFs measured in each individual for all targets was not correlated with breast tissue donor age (a), but the sum of *PIK3CA* and *TP53* MFs for each individual was significantly correlated with tissue donor age (b). *PIK3CA* and *TP53* were analyzed because they had previously been identified as containing hCDMs prevalent in breast cancers (Harris *et al*., 2019). Figure 7 also illustrates how tissue donor age might be related to breast cancer risk; cumulative age‐related risk was calculated from data in the SEER database and plotted relative to age (c) and the sum of MFs measured at each tissue donor age is plotted relative to the calculated breast cancer risk at that age (d).

### 3.5 Analysis of clonal expansion

Analysis of absolute deviation from a population median is an approach for assessing clonal expansion, including the analysis of the median or mean absolute deviation. Median and mean absolute deviations in MFs measured in each breast sample were calculated and related to tissue donor age, to determine whether clonal expansion increased with age as expected. Considering all panel targets, median absolute deviation in MFs ≥10^−4^ did not correlate significantly with tissue donor age for lung (Pearson *r* = 0.5321, P [one‐tailed] = 0.0702) or breast (*r* = 0.2605, P (one‐tailed) = 0.2492] (Figure S8a) and subsequent analyses failed to identify a subset of targets that correlate with lung tissue donor age. For breast, the median absolute deviation of *PIK3CA* and *TP53* MFs ≥10^−4^ correlated significantly with tissue donor age (Pearson *r* = 0.6329, P [one‐tailed] = 0.0337) (Figure S8b). Mean absolute deviation was also examined and the breast specific targets showed a better correlation with age than all targets, but neither was significantly correlated with age (Figure S8c,d).

## 4 DISCUSSION

There is growing impetus in the scientific community to move away from the paradigm of carcinogenicity testing based on the RTB. This has prompted development of approaches to prioritize chemicals for carcinogenicity testing and the adoption of alternative carcinogenicity assessment strategies (Morton *et al*., 2012; Luijten *et al*., 2016; Yauk *et al*., 2019). Alternative in vivo mouse models have been developed that can speed carcinogenicity testing in this species (Donehower 1996; Flammang *et al*., 1997; Schwetz and Gaylor 1998; Spalding *et al*., 1999; Bourcier *et al*., 2015). Nevertheless, current strategies for carcinogenicity testing require additional improvement, specifically better prediction of rodent tumor responses from shorter‐term endpoints and strengthening of the scientific knowledge underpinning rodent to human extrapolation (Zeiger and Stokes 1998).

The use of hCDMs as quantitative biomarkers of carcinogenic effect is a promising approach for improving carcinogen testing because quantifying CDMs has the potential to capture information on clonal expansion of cells with neoplastic potential, at a very early stage in the carcinogenic process. Our previous work documented how specific CDMs accumulate in treated rodent tissues following short‐term exposures to carcinogens and examined the impact of dose and exposure duration (Verkler *et al*., 2008; Meng *et al*., 2010; McKinzie and Parsons 2011; Wang *et al*., 2012). Importantly, we reported that the tissue‐specific carcinogenic impact of hCDMs is related to a metric based on interindividual variability in normal tissue levels (Parsons *et al*., 2017). We interpret this as meaning that the magnitude of interindividual variability in CDMs is reflective of clonal expansion driven by CDMs in individuals, which occurs in a stochastic manner that mirrors the carcinogenic process itself (Harris *et al*., 2019). Another way to state this is that the more heterogeneity there is in normal tissue, the more potential there is for clonal selective advantage leading to carcinogenesis. Consistent with this, it was shown in treated rodents that a metric based on treatment group variation in CD MF correlated with tumor response better than a metric based on treatment group CD geometric mean MF (Parsons 2018).

It is now understood that CDMs are prevalent in normal human tissues (Sudo *et al*., 2006; Gao *et al*., 2009; Parsons *et al*., 2010; Myers *et al*., 2014a; Martincorena *et al*., 2015; Myers *et al*., 2015; Young *et al*., 2016; Parsons *et al*., 2017; Martincorena *et al*., 2018; Suda *et al*., 2018; Salk *et al*., 2019; Yokoyama *et al*., 2019), leading one to ask how such mutations can be used as biomarkers if they are so universally present. Variability in CDM frequency across a homogeneous group of individuals (i.e., individuals of the same age and sex) or a rodent treatment group can be used to assess clonal expansion of cells carrying CDMs, which is viewed as a functional indicator of carcinogenic potential and/or effect, respectively (see Figure 1).

Building on past efforts to develop hCDMs as quantitative biomarkers of cancer risk, here we describe development and characterization of an EC‐NGS method, CarcSeq, for the analysis of a human amplicon panel encompassing many hCDMs. This was conducted in human based on the idea it is first necessary to identify human hCDMs that are relevant biomarkers before identifying which conserved rodent hCDMs have similar tissue‐specificity. Additionally, human samples that had been analyzed previously for CDMs by ACB‐PCR were available for cross‐platform validation (Parsons *et al*., 2017).

CarcSeq is similar to the Safe‐Sequencing System (Safe‐SeqS) (Kinde *et al*., 2011) or AmpliSeq HD technology (Thermo Fisher Scientific). Safe‐SeqS relies on UMIs incorporated into primers or endogenous UMIs, whereas AmpliSeq HD relies on UMIs incorporated into primers. Specifically, Safe‐SeqS uses two cycles in the first‐round of PCR to assign UMIs to an amplicon. This limits its application in situations wherein multiple amplicons need to be analyzed from a limited sample. Furthermore, two PCR cycles could be limited in efficiency to assign UMIs due to presence of chemical fixatives in clinical samples (Kinde *et al*., 2011). Our CarcSeq approach used 38 cycles to assign UMIs to amplicons during first‐round of multiplex PCR. While the assignment of multiple UMIs to the same molecule may occur during the first round of PCR in CarcSeq, we ameliorated this concern by diluting the input that goes into the final PCR amplification of the sample enrichment step to 1–1.5 million molecules and requiring three mutant molecules to construct a SSCS (so molecules amplified in the earliest cycles are enriched in the sequenced population).

EC‐NGS methodologies depend on the use of UMIs to identify replicate sequence reads from the same template molecule, allowing genetic variants shared across reads to be identified as mutations and those not shared to be identified as sequencing or PCR errors. Incorporation of UMIs as part of a primer sequence is an efficient strategy for correcting sequencing or late PCR errors, but may not correct errors that occur within the first few cycles of PCR (Salk *et al*., 2018). However, in the initial PCR amplification of our CarcSeq approach, we employed the same high‐fidelity PCR conditions used for ACB‐PCR, which has a sensitivity of 10^−5^, suggesting that the background error rate from early PCR errors would be in that range.

We employed a SSCS approach requiring three sequences with the same UMI to comprise a SSCS. In contrast, DS requires six sequences with the same UMI to comprise a duplex consensus sequence (DCS) to maximize DS efficiency (Kennedy, 2014). Because DS has a lower consensus‐making efficiency than a SSCS approach, DS requires greater read depths to construct the same number of consensus sequences (Salk *et al*., 2018). Thus, SSCS approaches have the potential to be more cost effective. That said, DS undoubtedly produces fewer background errors and, therefore, is expected to be more sensitive, provided enough DCS are assembled to take advantage of that sensitivity and avoid false negatives. CD MF levels are relatively large compared with other types of mutations (i.e., compared with somatic germ cell or neutral somatic mutations), so the analysis of hCDMs is an application where error‐correction by SSCS is a viable option.

Accurate assessment of the extent of clonal expansion across different targets within different DNA samples presents challenges with respect to rare event quantification. If one is using an EC‐NGS approach to describe a mutational signature, then all mutations (even those that are detected only once) are of interest. To develop a metric of clonal expansion in response to a carcinogenic exposure, accurate quantification of MF is impacted by both the numerator (number of mutant consensus sequences detected) and the denominator (the number of consensus sequences assessed). Specifically, due to sampling errors, the accuracy of a MF measurement will be degraded by the quantification of MFs based on the detection of a single mutant molecule (one SSCS or DCS). It has been estimated that if the true MF in a population is 1/X then the mutation will go undetected (MF = 0) in ~one‐third of replicate measurements on a population of size X and a MF of ≥2/X will be reported in ~one‐third of replicate measurements, which correspond to sampling errors of the infinite amount or ≥200% of the true MF (McKinzie *et al*., 2001). In rare event detection, one way to achieve greater precision is to require measurements be based on the detection of more than one molecule/SSCS. In our post‐sequencing analyses, we required detection of at least three SSCSs for further consideration as a “measured MF.” If 3/X is the true MF in a sample of size X, then in ~one‐third of replicate samples the mutation would be measured as 2/X and in ~one‐third of replicate samples the mutation would be measured as ≥4/X, which means most sampling errors will be of a magnitude of ±30%. Imprecision due to sampling errors will deteriorate the power to detect significant differences in clonal expansion between populations/treatment groups.

To further increase the power of the CarcSeq approach to yield a metric of clonal expansion, we employed a filter to eliminate invariant MF measurements. In the current study, specific mutations/positions where the COV across 9 normal samples was <60% were removed from each data set. This also helped to remove pseudogene artifacts and SNPs. The pseudogene artifacts were caused by either primers annealing to highly‐homologous, pseudogenes sequences that include SNVs compared with the true CD genes or sequence misalignment, rather than due to the detection of a mutant subpopulations (Figure S4d). Two types of data were collected to justify COV filtering: (a) visualizing the COV of ACB‐PCR concordant mutations, SNPs and pseudogene artifact identified bioinformatically (through homologies at nontarget sites where large numbers of reads aligned) and (b) ddPCR results confirming that some mutations were not present in the original genomic DNA samples (Figures S4 and S5).

Although the robustness and broad applicability of the use of 60% as a cutoff with the COV approach will require further investigation, in the current work the CarcSeq measurements were validated in several ways. The mutational profiles for normal and tumor were compared in terms of: (a) numbers of mutations detected, (b) significant differences in geometric mean MF, and (c) significant differences in variance. Assuming the approach accurately captured MF measurements, one would expect to see significantly greater geometric mean MFs and significantly greater variance in carcinomas than in normal tissues. These criteria for validation were observed for lung MF measurements ≥10^−5^ and COV ≥60% and for all breast MFs ≥10^−4^ (Table 4). The fact that greater significance in MF measurements ≥10^−5^ was observed for lung than for breast may be related to the overall larger numbers of SSCSs in the lung data set. Variance is considered the more important criteria for validation because it reflects the extent of clonal expansion. Thus, breast and lung measurements ≥10^−4^ and COV ≥60%, which also demonstrated significant differences in variance between normal and tumor, also validate the CarcSeq approach.

Clonal expansion of hotspot mutations was visually apparent in the data, specifically clonal expansion of *PIK3CA* mutations in multiple ductal carcinoma samples (Figure 3) and clonal expansion of *EGFR*, *KRAS*, and *TP53* mutations in multiple lung adenocarcinoma samples (Figure 4). Indeed, the fact that these clonal expansions are occurring in expected drivers with respect to tissue type is internal validation of CarcSeq MF measurements. Consistent with known COSMIC *STK11* tumor‐specificity (mutations present in 11% of lung adenocarcinomas but only 1% of ductal carcinomas), relatively high levels of *STK11* mutations were observed in lung, but not breast samples. However, these mutations appeared at similar levels in lung adenocarcinomas and normal lung samples (Figure 4). How this related to CarcSeq methodology or the quality of the data sets analyzed is currently unclear and additional studies will be needed to clarify which hCDMs will be informative predictors of future lung cancer development.

Quantitatively, some of the differences in hotspot MFs between normal and tumor were smaller than perhaps expected. This may be due to one potential variable that was not controlled in this study; specifically, a potential limitation of the current study is that different numbers of diploid cell equivalents were evaluated. Overall, large samples were analyzed relative to the sensitivity of the assay; most DNA samples were derived from 10^7^ to 10^8^ diploid cell equivalents, although one ductal carcinoma sample and one lung adenocarcinoma sample comprised only ~10^6^ diploid cell equivalents. Because the CarcSeq method detected MFs ≥10^−4^, most of the reported mutations are expected to have been represented by 1,000 or 10,000 s of mutant molecules. Somatic clones carrying CDMs may not be evenly distributed across normal tissues, as mutant subpopulations may not be evenly distributed within a tumor tissue sample. The probability of detecting clones will be increased using larger tissue samples, but the quantitative impact of any particular clonal expansion may be diluted out in a large tissue sample. Future studies intended to assign a cancer risk to an individual based on a CDM biomarker should be performed using equal and fairly large tissue samples, because analysis of smaller numbers of cell equivalents may result in tissue sampling variability that could confound reproducible estimation of risk. Clearly, the question of tissue sample size in the analysis of clonal expansion by EC‐NGS requires further investigation.

CarcSeq sensitivity was investigated by comparison of CarcSeq and ACB‐PCR MF measurements (comparing analyses of CarcSeq MFs ≥10^−5^ or ≥10^−4^) and by a reconstruction experiment. When CarcSeq MF measurements ≥10^−4^ and COV ≥60% were compared with ACB‐PCR MF measurements, essentially no bias was observed between the two methods (Figure 5a) and the values correlated (Spearman r = 0.9371, Figure 5c). Less bias and stronger correlation was observed when only MFs ≥10^−4^ were examined as compared with MFs ≥10^−5^ (Figure 5a vs. b and Figure 5c vs. d, respectively), although the bias remains small and the correlation strong (Spearman *r* = 0.6784) using the 10^−5^ cutoff. In the reconstruction experiment, a near‐perfect correlation was observed between CarcSeq MF measurements ≥10^−4^ and the defined *PIK3CA* E545K MF standards (Pearson *r* = 0.9996, Figure 6a). When CarcSeq MF measurements <10^−4^ were included, the correlation was not as strong (Pearson *r* = 0.9832, Figure 6b), which suggests a CarcSeq background level of mutation ≥10^−5^.

More statistically‐significant comparisons of geomean MF were observed between normal and tumor using a MF cutoff 10^−4^ than 10^−5^. However, the observation that not all normal versus tumor geomean comparisons of MFs ≥10^−4^ were significant indicates geomean MF may not be the ideal metric to consider in CarcSeq analysis of CD clonal expansion. Conversely, significant increases in variance in all tumor versus normal comparisons using MFs ≥10^−4^ compared with normal tissue and lack of significance for some normal to tumor comparisons using MFs ≥10^−5^ suggests variance may be a useful metric of clonal expansion and CarcSeq sensitivity is ≥10^−4^. Thus, the totality of the data from the broad CarcSeq target and the single site reconstruction experiment are consistent with the interpretation that CarcSeq has a sensitivity between 10^−5^ and 10^−4^, and this agrees with previous reports of SSCS background error frequency of ~3.4 × 10^−5^ (Schmitt *et al*., 2012; McKinzie and Bishop 2020).

**FIGURE 8:**
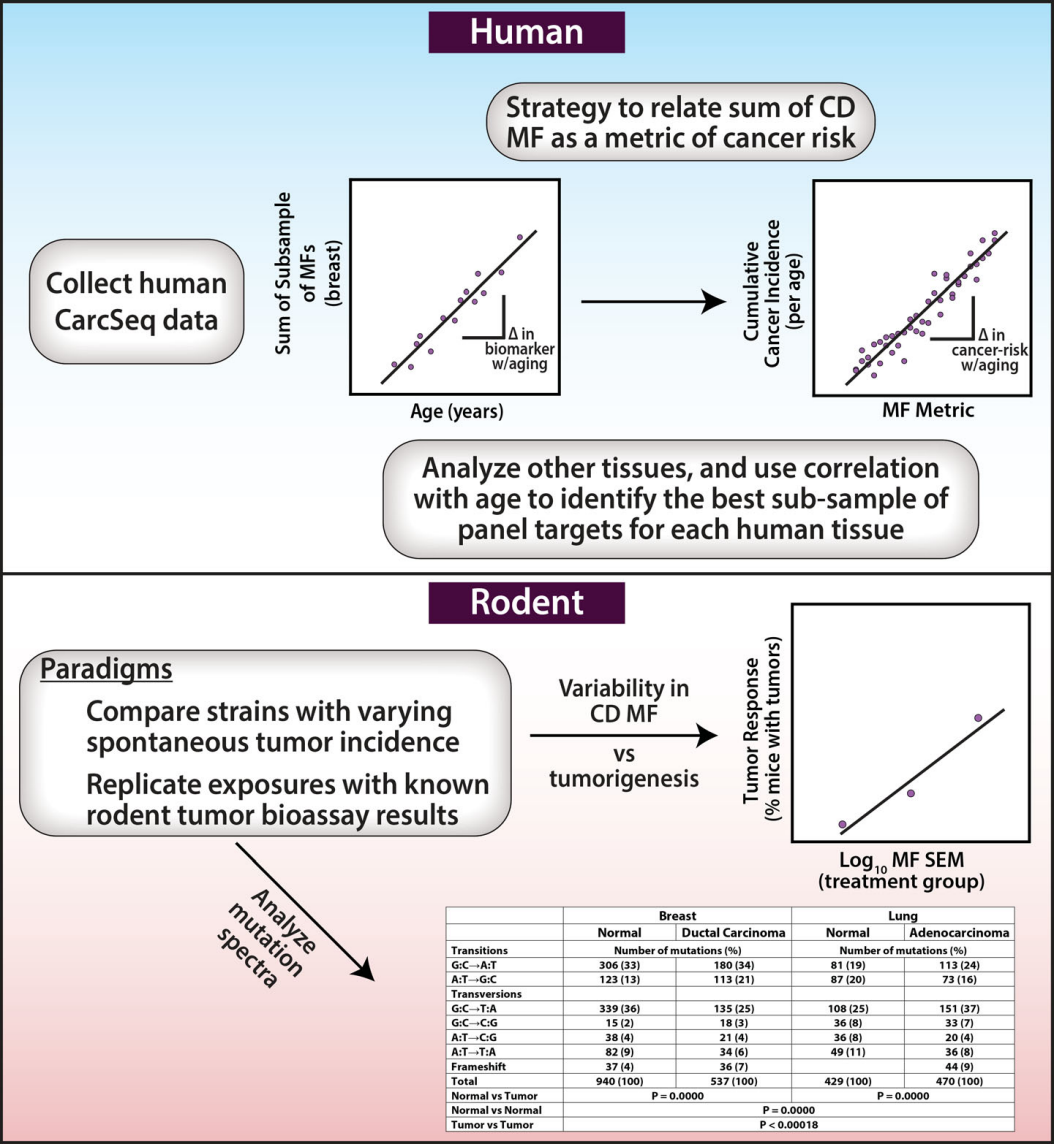
Depiction of experimental paradigms that could be used to relate tumor incidence in human or rodent to a metric based on analyzing batteries of hCDMs

A final approach for validating CarcSeq MF measurements, and the overall approach of using CD MF as a metric of cancer risk, was to correlate normal MF measurements with tissue donor age, because age is the single most important risk factor for cancer. The significant correlation observed between the sum of MFs in breast‐specific targets for individuals and age is encouraging, as was the initial attempt to examine the correlation between measures of clonal expansion with age (Figures 7 and S8). Obviously much more data are needed to establish measurements of specific CDMs as quantitative biomarkers of cancer risk for specific tumor types. Paradigms that should be utilized to develop such data, in both human and rodent, are illustrated in Figure 8.

In summary, we developed a novel method for EC‐NGS, called CarcSeq, and used it to quantify CD gene mutations in normal and malignant breast and lung samples. We propose using variability in CD MF as a metric and reporter of CD mutant cell clonal expansion. Incorporating this approach into preclinical drug development, as part of a battery of methods for carcinogenicity testing in rodent, would circumvent current limitations of the RTB. Clonal expansion of CDMs could be part of a battery of endpoints (with structural alerts, chemical class read‐across, transcriptomic data, and adverse outcome pathway analyses) used to improve the scientific basis of carcinogenicity assessment, particularly with respect to the identification of non‐genotoxic carcinogens (Kobets *et al*., 2019). Furthermore, measurements of the same predominant hCDMs in both rodent and human experimental paradigms (Figure 8) have the potential to provide a foundation for extrapolating from a carcinogen‐induced change in rodent CDM clonal expansion to an estimate of human cancer risk. Thus, CarcSeq provides a valuable opportunity to advance current cancer risk assessment and carcinogenicity testing practices.

## CONFLICT OF INTEREST

The authors declare they have no actual or potential competing financial interests.

## AUTHOR CONTRIBUTIONS

Barbara L. Parsons and Joshua Xu designed the study. Barbara L. Parsons and Meagan B. Myers acquired the tissues. Vijay Walia and Binsheng Gong developed the code and assembled an EC‐NGS pipeline. Meagan B. Myers and Vijay Walia developed ddPCR quantification of library preparations. Kelly L. Harris and Vijay Walia collected the CarcSeq data. Karen L. McKim and Kelly L. Harris performed ACB‐PCR and the CarcSeq reconstruction experiment, respectively. Barbara L. Parsons performed data analysis. All authors contributed to the preparation of the manuscript.

## Supporting information


**Appendix** **S1.** Supporting Information.
