# Supplementary material for: Quantification of cancer driver mutations in human breast and lung DNA using targeted, error‐corrected CarcSeq
Source: Environ Mol Mutagen. 2020 Sep 28;61(9):872–89. doi: 10.1002/em.22409 (PMC7756507; doi:10.1002/em.22409)
Supplement: Supplementary file 1 — Appendix S1. Supporting Information. [file EM-61-872-s001.docx]

Supplemental Material

**Quantification of Cancer Driver Mutations in Human Breast and Lung DNA Using Targeted, Error-corrected CarcSeq**

Kelly L. Harris^1,2^, Vijay Walia^1,3^, Binsheng Gong^4^, Karen L. McKim^2^, Meagan B. Myers^2^, Joshua Xu^4^, and Barbara L. Parsons^2,5^

^1^These authors contributed equally

^2^US Food and Drug Administration, National Center for Toxicological Research, Division of Genetic and Molecular Toxicology, 3900 NCTR Rd., Jefferson, AR 72079

^3^Current address: US Food and Drug Administration, Center for Devices and Radiological Health, 10903 New Hampshire Ave., Silver Spring, MD 20993

^4^Division of Bioinformatics and Biostatistics, National Center for Toxicological Research, 3900

NCTR Rd., Jefferson, AR 72079 Rd., Jefferson, AR 72079

^5^Correspondence to Barbara L. Parsons, Division of Genetic and Molecular Toxicology, National

Center for Toxicological Research, US Food and Drug Administration, HFT-120, 3900 NCTR

Rd., Jefferson, AR 72079

Barbara.parsons@fda.hhs.gov

Phone: 870-543-7946

**Table S1.** Primer sequences used for the amplification of amplicons.

| Gene | Hotspot Codon Mutations^†^ | Multiplex Group | Primer 1 Sequence | Primer 2 Sequence | Amplicon Length (bp) | Product Length (bp) |
| --- | --- | --- | --- | --- | --- | --- |
| *KRAS* | G12  G13 | Group 1 | 5’-(N9)-AAGGCCTGCTGAAAATGACTG-3’ | 5’-(N9)-AGCTGTATCGTCAAGGCACT-3’ | 100 | 82 |
| *SETBP1* | D868  G870  I871^‡^ | Group 1 | 5’-(N9)-GAAATCACGCTGTCCCCTGT-3’ | 5’-(N9)-CCTCCTTCGGGATTCTGAGC-3’ | 144 | 126 |
| *TP53* | G245  R248  R249 | Group 1 | 5’-(N9)-GGCTCTGACTGTACCACCATC-3’ | 5’-(N9)-GGCAAGTGGCTCCTGACC-3’ | 136 | 118 |
| *PIK3CA* | H1047 | Group 1 | 5’-(N9)-ACTGAGCAAGAGGCTTTGGA-3’ | 5’-(N9)-TGCATGCTGTTTAATTGTGTGG-3’ | 126 | 108 |
| *TP53* | R273  P278  R282* | Group 2 | 5’-(N9)-TGCCTCTTGCTTCTCTTTTCCT-3’ | 5’-(N9)-ATTCTCTTCCTCTGTGCGCC-3’ | 133 | 115 |
| *EGFR* | T790 | Group 2 | 5’-(N9)-ATGGCCAGCGTGGACAAC-3’ | 5’-(N9)-TCTCTTTGTGTTCCCGGACAT-3’ | 145 | 127 |
| *TP53* | R175  C176  H179 | Group 3 | 5’-(N9)-GGCCATCTACAAGCAGTCACA-3’ | 5’-(N9)-CAACCAGCCCTGTCGTCTC-3’ | 142 | 124 |
| *STK11* | F354^‡^ | Group 3 | 5’-(N9)-CATGACTGTGGTGCCGTACT-3’ | 5’-(N9)-GCACCGTGAAGTCCTGAGT-3’ | 122 | 104 |
| *PIK3CA* | E542  E545 | Group 3 | 5’-(N9)-GACAAAGAACAGCTCAAAGCA-3’ | 5’-(N9)-ACAGAGAATCTCCATTTTAGCACT-3’ | 131 | 113 |
| *BRAF* | V600 | Group 3 | 5’-(N9)-TTTCCTTTACTTACTACACCTCAGA-3’ | 5’-(N9)-TGGATCCAGACAACTGTTCAA-3’ | 150 | 132 |
| *EGFR* | L858 | Group 4 | 5’-(N9)-CTGGTGAAAACACCGCAGC-3’ | 5’-(N9)-GCTGACCTAAAGCCACCTCC-3’ | 138 | 120 |
| *APC* | R1450 | Group 4 | 5’-(N9)-GACAAACCATGCCACCAAGC-3’ | 5’-(N9)-GCAGCTTGCTTAGGTCCACT-3’ | 148 | 130 |
| *NFE2L2* | D27^‡^ | Group 4 | 5’-(N9)-AGCCTTTTTCGCTCAGTTACAA-3’ | 5’-(N9)-GTACCTGGGAGTAGTTGGCAG-3’ | 131 | 113 |

*includes first position of codon R282

†hotspot codons identified in Harris et al. 2019

‡hotspot codons identified in COSMIC database

**Table S2.** Multiplex PCR reactions and cycling conditions.

| Multiplex Group 1 PCR Reaction | |
| --- | --- |
| 10 μM PIK3CA (H1047) forward primer  10 μM PIK3CA (H1047) reverse primer  10 μM TP53 (G245, R248, R249) forward primer  10 μM TP53 (G245, R248, R249) reverse primer  10 μM KRAS (G12, G13) forward primer  10 μM KRAS (G12, G13) reverse primer  10 μM SETBP1 (D868, G870, I871) forward primer  10 μM SETBP1 (D868, G870, I871) reverse primer  10X Pfu buffer  2.5 mM dNTPs  MgCl_2_  Pfu Ultra DNA polymerase | 4 μL  4 μL  4 μL  4 μL  4 μL  4 μL  6 μL  6 μL  24 μL  32 μL  8 μL  12 μL |
| Cycling conditions: 2 min denaturation at 94 °C, followed by 38 cycles of 1 min at 94 °C, 1 min at 52 °C, and 1 min at 68 °C, and a final 7 min extension at 68 °C. | |
| Multiplex Group 2 PCR Reaction | |
| 10 µM TP53 (R273, P278, R2782) forward primer  10 µM TP53 (R273, P278, R2782) reverse primer  10 µM EGFR (T790) forward primer  10 µM EGFR (T790) reverse primer  10X Pfu buffer  2.5 mM dNTPs  MgCl_2_  Pfu Ultra DNA polymerase | 4 μL  4 μL  7 μL  7 μL  24 μL  32 μL  6 μL  8 μL |
| Cycling conditions: 2 min denaturation at 94 °C, followed by 38 cycles of 1 min at 94 °C, 1 min at 50 °C, and 1 min at 65 °C, and a final 7 min extension at 68 °C. | |
| Multiplex Group 3 PCR Reaction | |
| 10uM STK11 (F354) forward primer  10uM STK11 (F354) reverse primer  10 µM PIK3CA (E542, E545) forward primer  10 µM PIK3CA (E542, E545) reverse primer  10 µM TP53 (R175, C176, H179) forward primer  10 µM TP53 (R175, C176, H179) reverse primer  10 µM BRAF (V600) forward primer  10 µM BRAF (V600) reverse primer  10X Pfu buffer  2.5 mM dNTPs  MgCl_2_  Pfu Ultra DNA polymerase | 10 μL  10 μL  4 μL  4 μL  4 μL  4 μL  4 μL  4 μL  24 μL  32 μL  10 μL  12 μL |
| Cycling conditions: 2 min denaturation at 94 °C, followed by 38 cycles of 1 min at 94 °C, 1 min at 50 °C, and 1 min at 65 °C, and a final 7 min extension at 65 °C. | |
| Multiplex Group 4 PCR Reaction | |
| 10uM NFE2L2 (D27) forward primer  10uM NFE2L2 (D27) reverse primer  10 µM APC (R1450) forward primer  10 µM APC (R1450) reverse primer  10 µM EGFR (L858) forward primer  10 µM EGFR (L858) reverse primer  10X Pfu buffer  2.5 mM dNTPs  MgCl_2_  Pfu Ultra DNA polymerase | 4 μL  4 μL  4 μL  4 μL  6 μL  6 μL  24 μL  32 μL  8 μL  12 μL |
| Cycling conditions: 2 min denaturation at 94 °C, followed by 38 cycles of 1 min at 94 °C, 1 min at 50 °C, and 1 min at 65 °C, and a final 7 min extension at 65 °C. | |


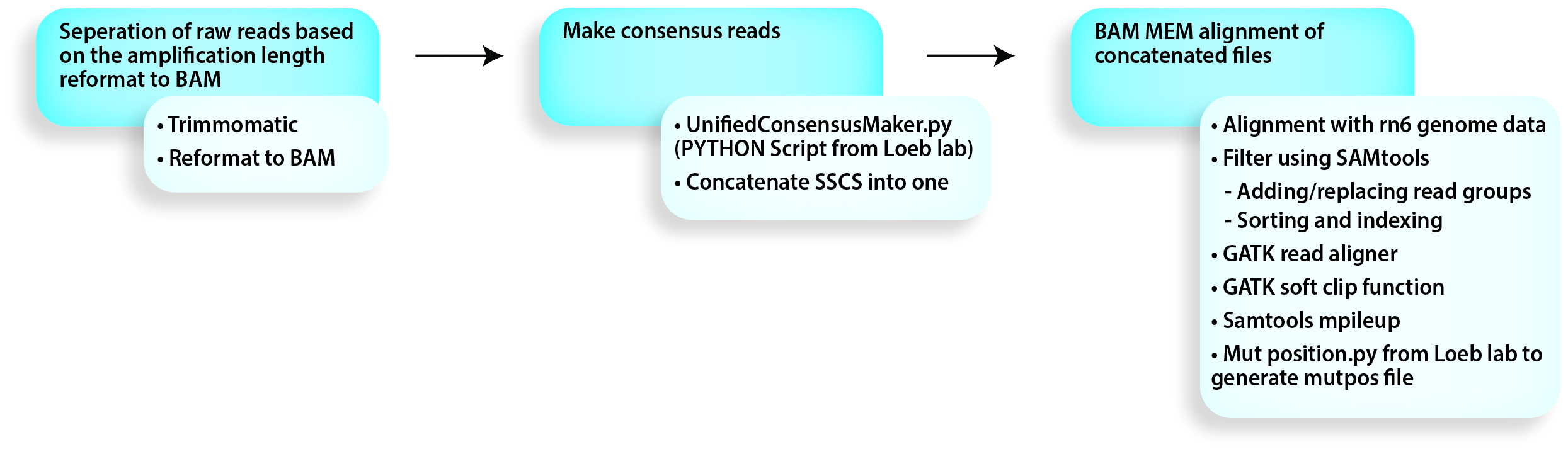


**Figure S1.** Steps in the bioinformatic analysis.

**Error Correction Sequencing Data Processing**

Reads from all four lanes were concatenated and analyzed using modified Trimmomatic tool to sort sequence reads by length of each amplicon (Bolger, A. M., Lohse, M., & Usadel, B. (2014). Trimmomatic: A flexible trimmer for Illumina Sequence Data. *Bioinformatics*, btu170). Picard tools (Broad Institute) were used to make compatible bam files for further analysis. A python script, UnifiedConsensusMaker.py, from Kennedy et al. (Kennedy, Schmitt et al. 2014) was modified to construct SSCSs based upon the 18 bp UMI sequences (nine bps combined from each end) for each amplicon, respectively. Output from above was concatenated for further alignment to human genome. At least three reads containing the same UMI were required to constitute a SSCS. Non-reference bases had to be present in at least 70% of reads within a SSCS for a variant call. Those in <70% of reads within a SSCS were not identified as mutant or reference (N calls by the python program). BWA-MEM alignment algorithm from Burrows-Wheeler Aligner package was used to aligned SSCSs to the GRCh38 human reference genome (Figure 2B). A suite of programs, Samtools (from htslib.org) was used to view, sort, and index referenced reads. Picard tools were used to add readgroups and were followed by indexing with Samtools. Local re-alignment was performed using RealignerTargetCreator and IndelRealigner from Genome Analysis Tool Kit (Broad Institute) using GRCh38 human reference genome. A pileup file was generated from the final SSCSs using mpileup function of Samtools. Python script, mut-position.py, from Kennedy et al. (Kennedy, Schmitt et al. 2014) was used to generate csv files. Non-classifiable variants were interpreted as PCR or sequencing errors. The pipeline generated two critical output files: 1) a comma-separated values (csv) file reporting median number of SSCSs representing each amplicon in a sample, considering all positions within an amplicon and 2) a mutation position (mutpos) csv file reporting the total number of mutants of each type detected (*i.e.*, numbers of T, C, G, A, insertion, or deletion mutations) and the number of unclassifiable SSCSs (Ns) for the total number of SSCSs representing each position/site within the target (depth).


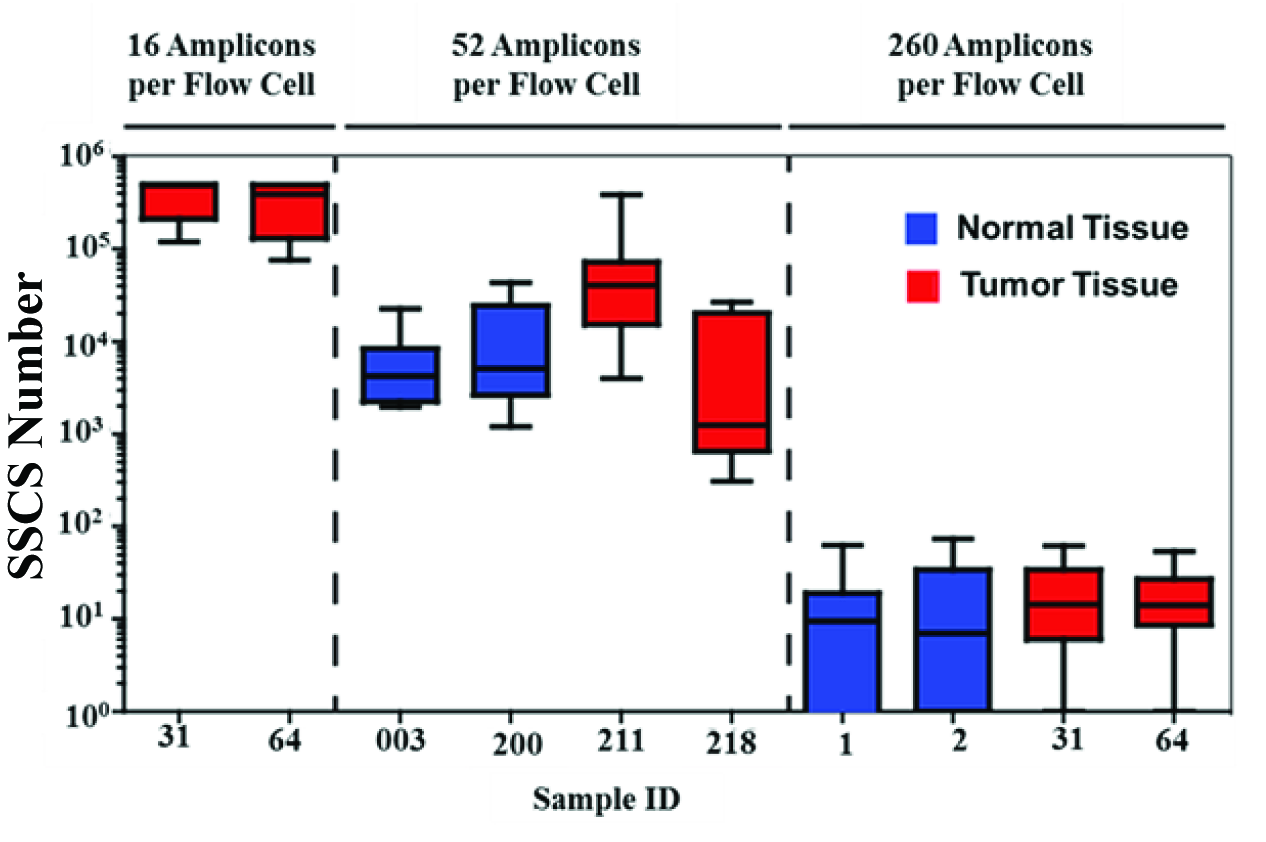


**Figure S2.** Relationship between numbers of SSCSs per amplicon and number of amplicons analyzed per Illumina mid-size flow cell. Note, this early experiment used the entire sample of adapted-ligated, gel-purified library prep as per the instruction of the Illumina TruSeq® ChIP Sample Preparation Kit, whereas all other data were collected using only 1 to 1.5 million input DNA copies of the representative *PIK3CA* H1047-containing amplicon as described in Methods, Library Preparation.


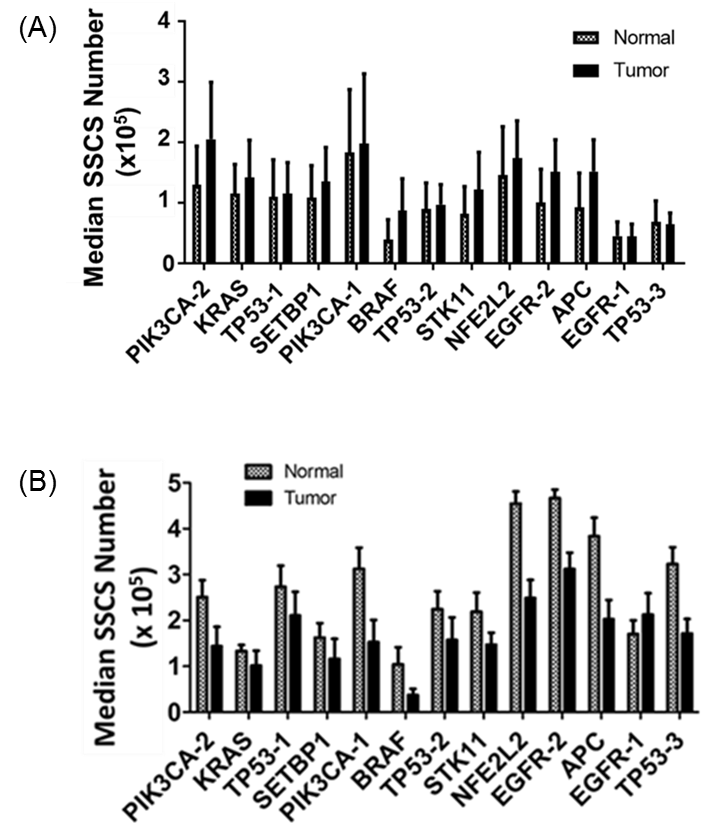


**Figure S3.** Distribution of SSCS numbers recovered for each amplicon in breast (A) and lung (B) samples.


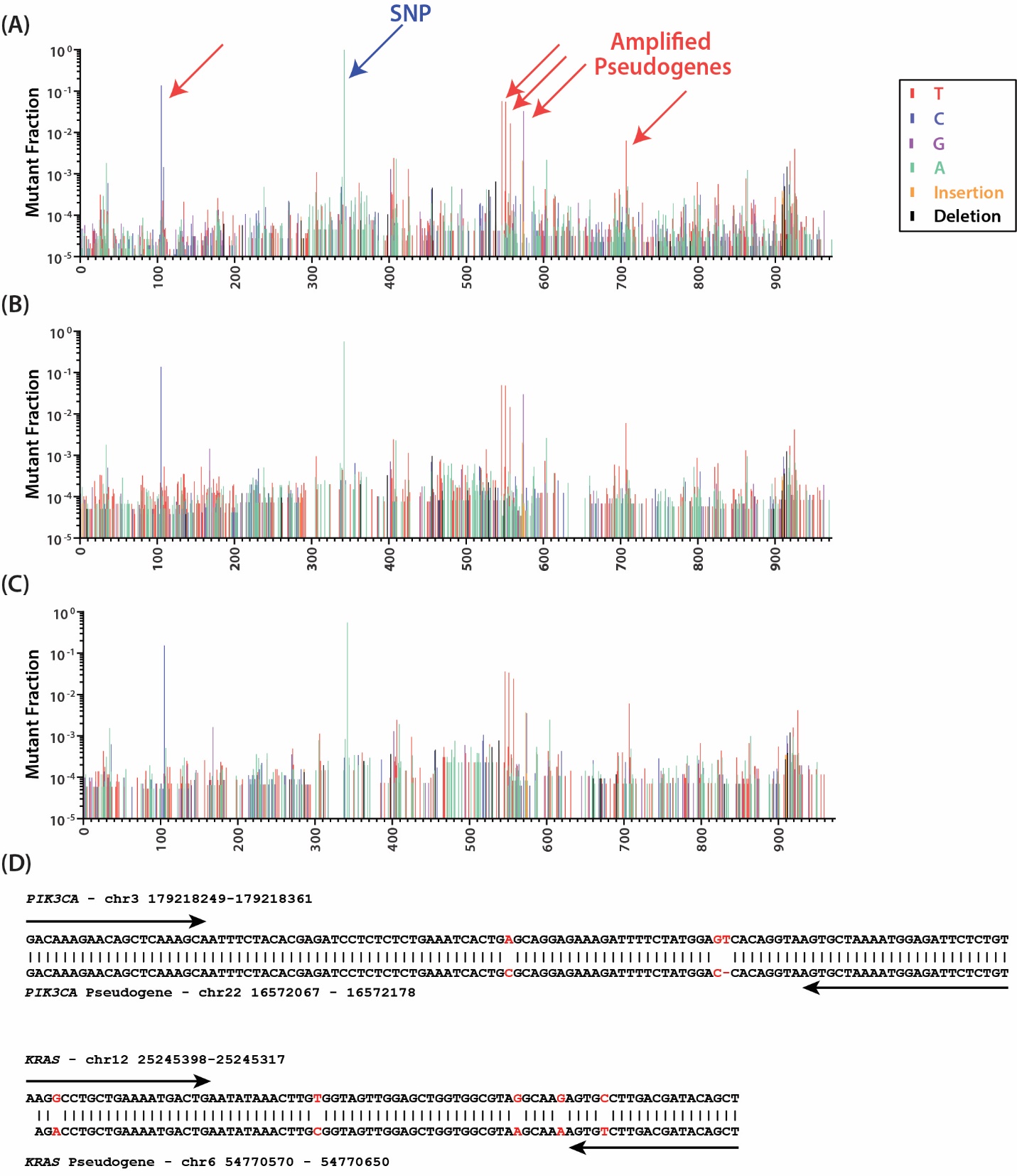


**Figure S4.** Representative profiles of three different normal lung samples with MFs ≥10^-5^, showing invariant peaks resulting from polymorphisms in pseudogenes, as well as an EFGR SNP (A-C). Mis-priming or misalignment of CarcSeq products with pseudogenes was identified as a source of invariant peaks (D). For example, the blue peak at position 105 (A→C) corresponds to a *PIK3CA* E545A artifacts not detected in genomic DNA. Two red peaks (T) between positions 550-560 correspond to *KRAS* pseudogene mismatches.


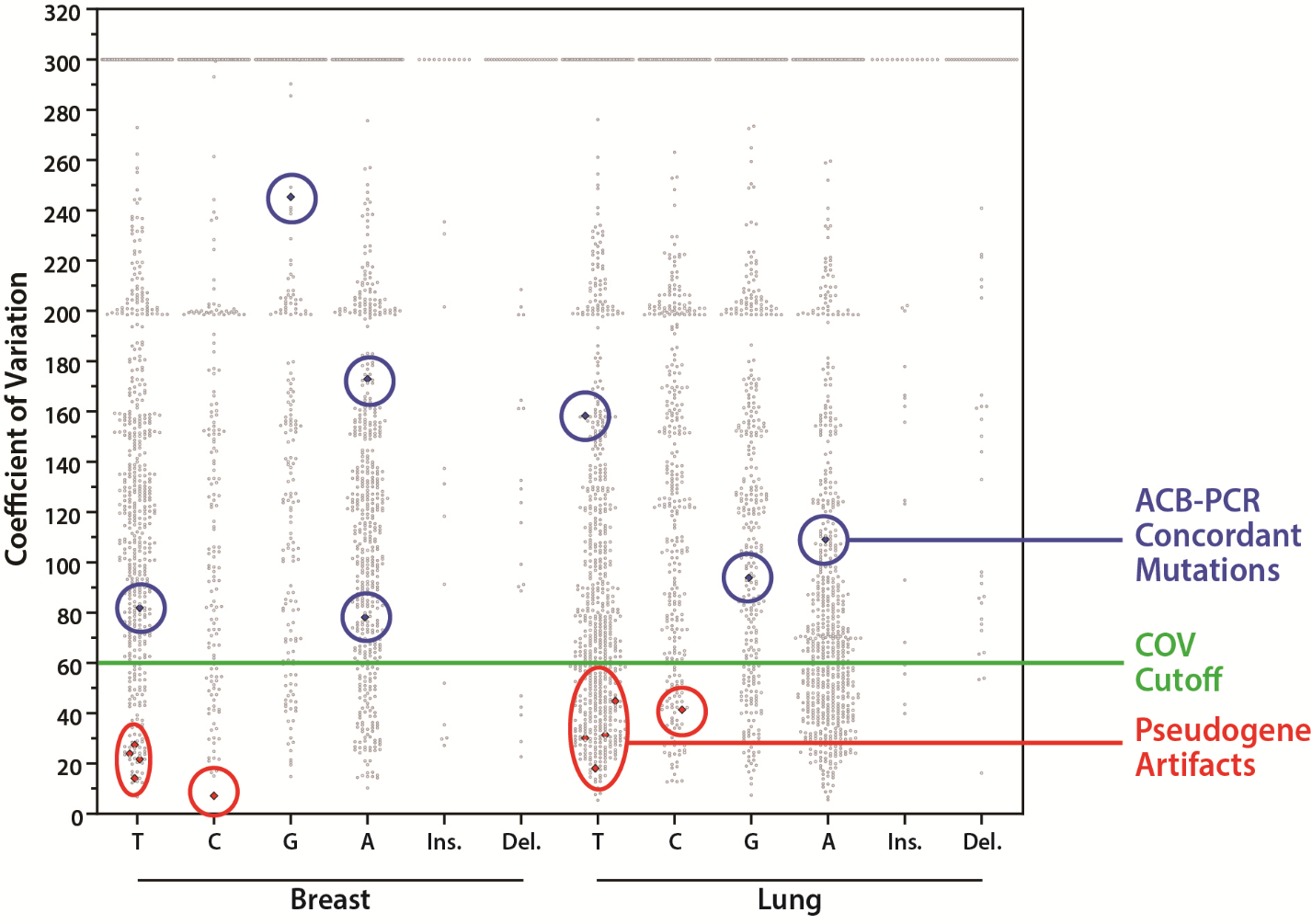


**Figure S5.** COV analysis for CarcSeq MF measurements of each type at each target position. The positions of CarcSeq and ACB-PCR concordant MF measurements are noted in blue. Bioinformatically-described pseudogene artifacts are indicated in red. The selected COV cutoff of 60 is denoted in green.


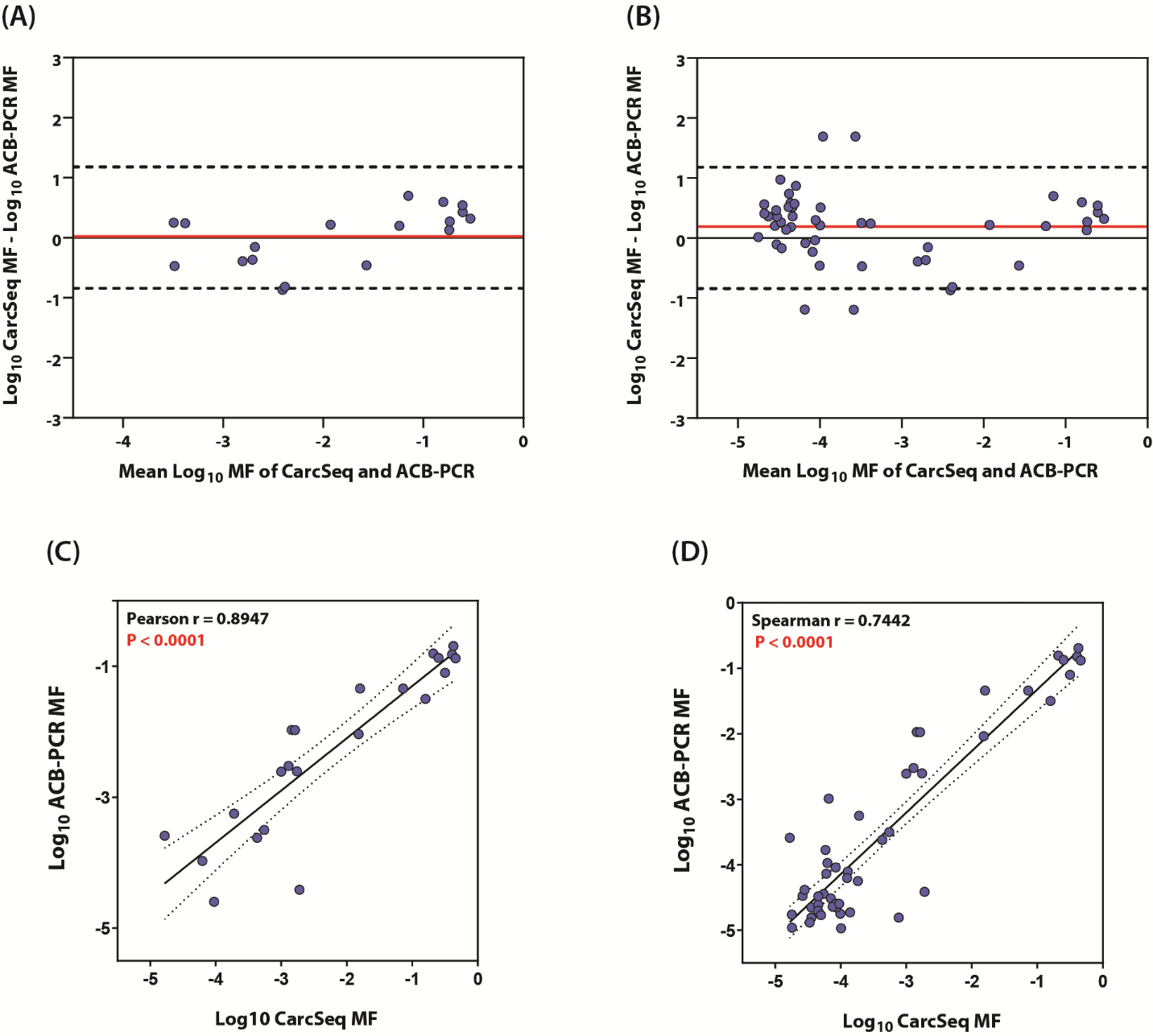


**Figure S6.** Comparison of CarcSeq and ACB-PCR measured MFs in combined normal breast and ductal carcinoma samples. Bland-Altman analysis was used to analyze bias between CarcSeq and ACB-PCR MF measurements ≥10^‑4^ (A) or ≥10^-5^ (B). Linear regression analysis of CarcSeq and ACB-PCR measured MFs >10^-4^ or >10^-5^ are shown in (C) and (D), respectively.


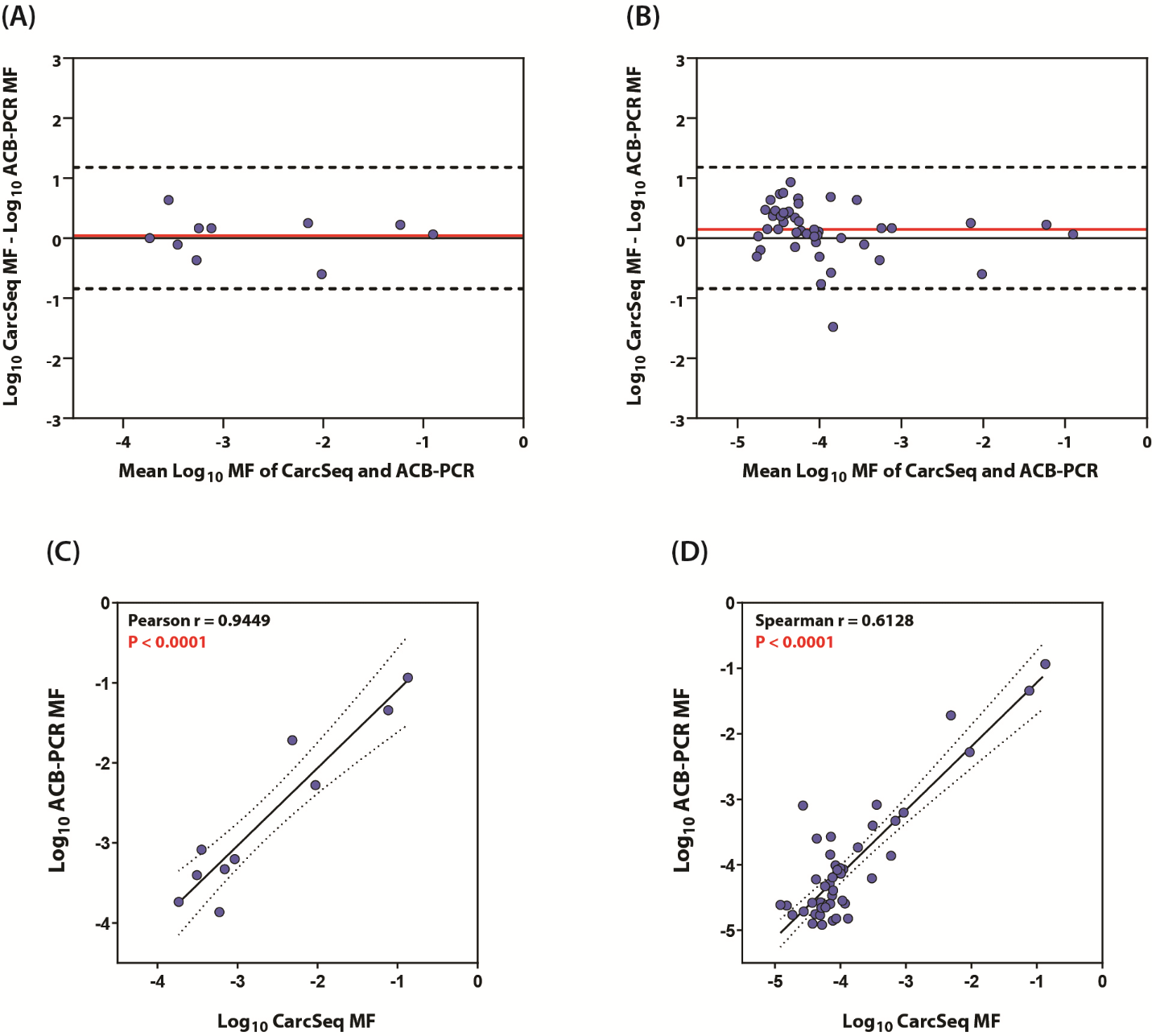


**Figure S7.** Comparison of CarcSeq and ACB-PCR measured MFs in combined normal lung and lung adenocarcinoma samples. Bland-Altman analysis was used to analyze bias between CarcSeq and ACB-PCR MF measurements ≥10^‑4^ (A) or ≥10^-5^ (B). Linear regression analysis of CarcSeq and ACB-PCR measured MFs >10^-4^ or >10^-5^ are shown in (C) and (D), respectively.

**
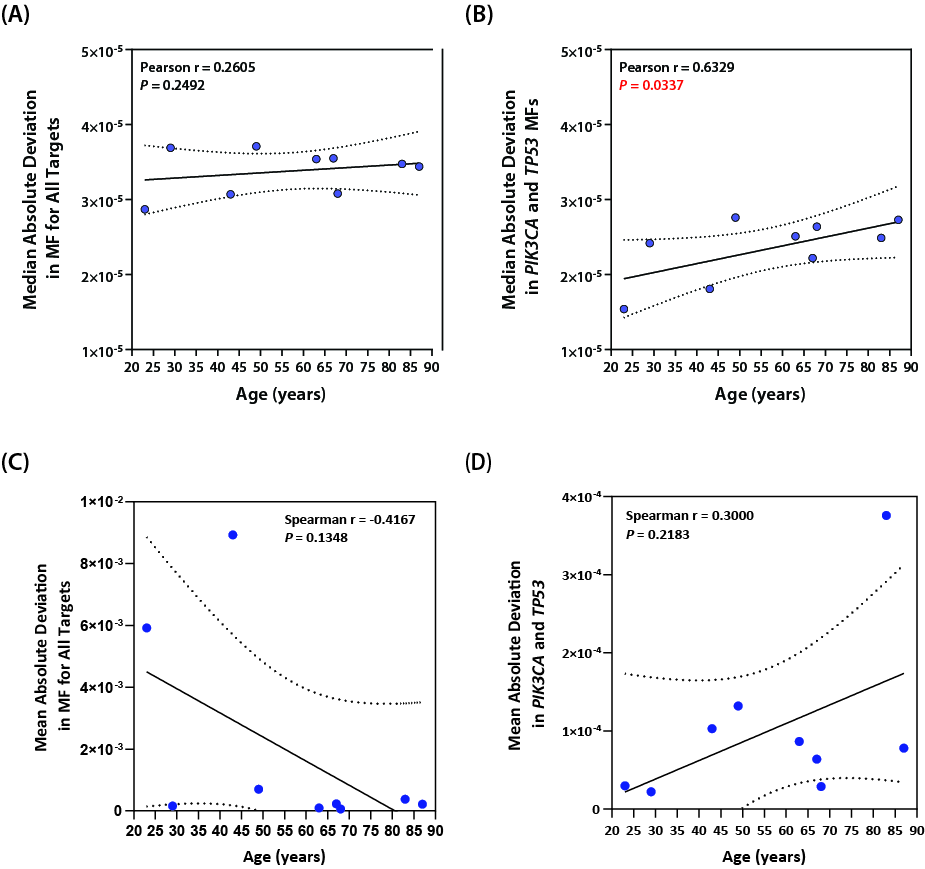
**

**Figure S8.** Correlations between median absolute deviation or mean absolute deviation and tissue donor age. The median MF and the absolute deviation between the median and each MF measurement was calculated for each normal breast sample. Then, considering MFs at all targets (A) or only *PIK3CA* and *TP53* MFs (B), the median absolute deviation was plotted relative to tissue donor age. Considering MF at all targets (C) or only *PIK3CA* and *TP53* MFs (D), the mean absolute deviation was plotted relative to tissue donor age. All tests are one-tailed.
